# Bi-allelic variants in *FSD1L* cause retinitis pigmentosa with or without neurological involvement

**DOI:** 10.1016/j.ajhg.2026.01.015

**Published:** 2026-02-19

**Authors:** Siying Lin, Francesca Cancellieri, Yexuan Cao, Andrew J. Lotery, Abigail R. Moye, Veronika Vaclavik, Fabienne Perren, Andrzej B. Poplawski, Elena R. Schiff, Mukhtar Ullah, Ana Belen Iglesias-Romero, Karolina Kaminska, Aleksandr Jestin, Marc Folcher, Sandrine Wallerich, Mariana M. Ribeiro, Vincent Hahaut, Simone Picelli, Debarshi Mustafi, Aleksander Tworak, Roman Smidak, Yumei Li, Jiaxiong Lu, Meng Wang, Omar A. Mahroo, Shyamanga Borooah, Mathieu Quinodoz, Krzysztof Palczewski, Andrew R. Webster, Carlo Rivolta, Rui Chen, Gavin Arno

**Affiliations:** 1 Division of Evolution, Infection and Genomics, School of Biological Sciences, Faculty of Biology, Medicine and Health, University of Manchester, Manchester, UK; 2 Manchester Centre for Genomic Medicine, Saint Mary’s Hospital & Department of Ophthalmology, Manchester Royal Eye Hospital, Manchester University NHS Foundation Trust, Manchester, UK; 3 National Institute of Health Research Biomedical Research Centre at Moorfields Eye Hospital and the UCL Institute of Ophthalmology, London, UK; 4 UCL Institute of Ophthalmology, University College London, London, UK; 5 Institute of Molecular and Clinical Ophthalmology Basel (IOB), Basel, Switzerland; 6 Department of Ophthalmology, University of Basel, Basel, Switzerland; 7 Department of Ophthalmology and Visual Sciences, Gavin Herbert Eye Institute – Robert M. Branson Center for Translational Vision Research, University of California, Irvine, Irvine, CA, USA; 8 Southampton Eye Unit, University Hospital Southampton, Southampton, UK; 9 Faculty of Medicine, University of Southampton, Southampton, UK; 10 Jules-Gonin Eye Hospital, Fondation Asile des Aveugles, University of Lausanne, Lausanne, Switzerland; 11 Department of Sciences and Medicine, LUNIC Laboratory, University of Fribourg, Fribourg, Switzerland; 12 Department of Clinical Neurosciences, Neurocenter of Geneva, LUNIC Laboratory, Geneva, Switzerland; 13 Division of Research, Greenwood Genetic Center, Greenwood, SC, USA; 14 Department of Ophthalmology, University of Washington, Seattle, WA, USA; 15 Division of Ophthalmology, Seattle Children’s Hospital, Seattle, WA, USA; 16 Department of Ophthalmology, St Thomas’ Hospital, London, UK; 17 Department of Ophthalmology, Shiley Eye Institute, University of California, San Diego, La Jolla, CA, USA; 18 Department of Genetics and Genome Biology, University of Leicester, Leicester, UK; 19 Department of Physiology and Biophysics, School of Medicine, University of California, Irvine, Irvine, CA, USA; 20 Department of Chemistry, University of California, Irvine, Irvine, CA, USA; 21 Department of Molecular Biology and Biochemistry, University of California, Irvine, Irvine, CA, USA

**Keywords:** inherited retinal dystrophy, retinitis pigmentosa, FSD1L, cilia, genome sequencing, retinal transcriptomics, single-cell transcriptome, splice variant, retina-enriched isoform, nanopore sequencing

## Abstract

Retinitis pigmentosa (RP) is an inherited retinal disease (IRD) characterized usually by progressive photoreceptor degeneration, leading to night blindness, peripheral visual field loss, and can progress to central vision impairment in some individuals. Despite advances in genomic diagnostics, many individuals with RP remain without a molecular diagnosis. We identified bi-allelic ultra-rare variants in fibronectin type II and Spry domain-containing protein 1-like (*FSD1L*) in six individuals with RP with or without neurological features from four unrelated families. *FSD1L* encodes a cytoplasmic protein, variants of which have not previously been associated with Mendelian disease. The gene is expressed in both human and mouse retinas that are enriched in cone and rod photoreceptors. Immunofluorescence and ultrastructure expansion microscopy show that FSD1L localizes along the photoreceptor microtubule axoneme, including the connecting cilium and outer segment, supporting a possible role in intracellular trafficking. A retina-enriched isoform of *FSD1L* includes an alternatively spliced exon (exon 10b), which we characterize as absent in minigene assays and affected individual-derived lymphocytes due to a deep intronic 26 nt deletion. Together, these findings support the association between bi-allelic disruption of *FSD1L* and IRD.

## Teaser

Bi-allelic ultra-rare variants in *FSD1L* are identified as a cause of retinitis pigmentosa. One variant identified disrupts the splicing of a retina-enriched isoform. FSD1L localizes to the photoreceptor axoneme, implicating disruption of intracellular trafficking as a possible mechanism for photoreceptor degeneration.

## Main text

Inherited retinal disease (IRD) describes a clinically and genetically heterogeneous group of disorders characterized typically by progressive retinal degeneration, leading to visual impairment and blindness. IRDs are a leading cause of vision loss among children and working-age adults and affect an estimated 5.5 million individuals worldwide,^1^^,^^2^ representing a significant global healthcare burden.

Retinitis pigmentosa (RP [MIM: 268000]) is the most common form of IRD, affecting approximately 1 in 3,500 individuals in the USA and Europe.^3^^,^^4^ RP typically presents with nyctalopia (night blindness), followed by progressive peripheral visual field loss, and, in many cases, progresses to central visual impairment. RP, and IRDs more generally, may occur as an isolated disease, limited to ocular involvement, or as part of syndromic conditions involving additional tissues, organs, or systems, including hearing loss, neurodevelopmental disease, or metabolic dysfunction.^5^

Despite advances in high-throughput sequencing technologies and diagnostic pipelines, up to 40%–50% of IRD-affected individuals remain molecularly undiagnosed,^6^ limiting access to accurate prognostic information, genetic counseling, and emerging gene-directed therapies. This paucity of data suggests that novel mechanisms of degenerative retinal disease remain to be identified.

Here, we report the clinical and genetic findings of six affected individuals, two males and four females aged 14–51 years, from four unrelated families. All six individuals manifested RP, while some had additional clinical findings, as summarized in Table 1 (further details are available in the supplemental notes: clinical findings). All six individuals had bi-allelic ultra-rare variants (AF [allele frequency] < 0.00015; gnomAD v.4.1.0) in *FSD1L* (MIM: 609829), which encodes for the fibronectin type II and Spry domain-containing protein (FSD)1-like (FSD1L) protein. Variants were identified by exome sequencing or genome sequencing (GS) analysis performed as part of large-scale projects aimed at elucidating the etiology of genetic disease, including the UK 100,000 Genomes Project (individuals A.III-1 and A.III-3), the UK National Health Service (NHS) Genomic Medicine Service (GMS) (individual B.II-1),^6^^,^^7^ and ongoing research studies at the University of California, Irvine (UCI), and the University of Washington (individuals C.II-1 and C.II-2) and the Institute of Molecular and Clinical Ophthalmology, Basel (individual D.II-2).

**Table 1:** Clinical features for individuals with bi-allelic *FSD1L* variants (GenBank: NM_001145313.3)

**Family**	**A (GC4822)**		**B (GC17709)**	**C**		**D (CHlaus0427)**
Individual	A.III-1	A.III-3	B.II-1	C.II-1	C.II-2	D.II-2
Allele 1	c.1049G>A (p.Arg350Gln)	c.1049G>A (p.Arg350Gln)	c.488G>A (p.Arg163His)	c.488G>A (p.Arg163His)	c.488G>A (p.Arg163His)	c.1037_1038delinsT (p.Pro346Leufs^∗^8)
Allele 2	c.1428del (p.Phe476Leufs^∗^22)	c.1428del (p.Phe476Leufs^∗^22)	c.745C>T (p.Arg249^∗^)	c.226_227del (p.Ser77Argfs^∗^4)	c.226_227del (p.Ser77Argfs^∗^4)	c.1025+624_1025+649del
Country of recruitment	UK	UK	UK	USA	USA	Switzerland
Ethnicity	White British	White British	White British	White American	White American	White Swiss
Sex	male	female	female	female	male	female
Age at last examination	36 years	32 years	32 years	14 years	12 years	51 years
Diagnosis	RP with mild neurological involvement	RP with possible mild neurological involvement	RP, no known neurological involvement	RP, no known neurological involvement	RP, no known neurological involvement	RP, no neurological involvement

**Ocular features**						

Initial symptoms (age of onset)	nyctalopia (9 years)	nyctalopia (13 years)	nyctalopia (5 years)	nyctalopia (14 years)	asymptomatic (diagnosed after sibling diagnosis)	nyctalopia, high myopia (10 years)
BCVA (Snellen)	OD: 20/40 OS: 20/200	OD: 20/120 OS: 20/200	OD: 20/50 OS: 20/80	OD: 20/50 OS: 20/50	OD: 20/32 OS: 20/32	OD: 20/1,200 OS: 20/400
Refraction	OD: +0.50/−3.50 × 11° OS: +0.25/−3.50 × 176°	OD: +0.75/−2.75 × 16° OS: 1.00/−5.75 × 147°	emmetropia	emmetropia	emmetropia	OD: −0.5/−2.25 × 63° OS: −0.75/−1 × 43° (pseudophakic)
Lens status	phakic with clear lenses bilaterally	bilateral posterior subcapsular lens opacities	bilateral pseudophakia (cataract surgery OD 30 years; OS 27 years)	bilateral pseudophakia (cataract surgery OD 13 years; OS 13 years)	phakic with clear lenses bilaterally	bilateral pseudophakia (cataract surgery OU 36 years)
Fundus	hypopigmented fundus with prominent choroidal vasculature, macular atrophy, pale optic discs, attenuated vessels, no pigmentary retinopathy	hypopigmented fundus with prominent choroidal vasculature, pale optic discs, and attenuated vessels, pigmentary retinopathy	pale optic discs, attenuated retinal vessels, and mid peripheral pigmentary retinopathy	bone spicule-type pigmentation superonasally, normal optic discs, loss of foveal reflex with macular edema bilaterally	normal fundus except for loss of foveal reflex with macular edema bilaterally	hypopigmented posterior pole macular atrophy, pale optic discs, attenuated vessels, pigmentary retinopathy
Autofluorescence	small hyper-AF ring at the macula	mid-peripheral hypo-AF with a central ring of hyper-AF	widespread hypo-AF with small central macular hyper-AF	hypo-AF peripherally without central hyper-AF	increased AF at the posterior pole with a hyper-AF ring at the macula	mid-peripheral and posterior pole hypo-AF, area of normal AF within arcades
OCT	mild ERM; perifoveal EZ loss with subfoveal sparing; no CME	perifoveal EZ loss with subfoveal sparing; no CME	perifoveal EZ loss with subfoveal sparing; no CME	perifoveal EZ loss with subfoveal sparing; CME	perifoveal EZ loss with subfoveal sparing; CME	thinning of retinal layers, EZ not visible, no CME
ERG (age of examination)	consistent with rod-cone dystrophy with marked macular involvement (19 years)	consistent with rod-cone dystrophy with marked macular involvement (15 years)	severely reduced rod and cone responses (16 years)	NP	NP	undetectable scotopic and photopic ERGs (42 years)
Visual fields	severely constricted to confrontation	severely constricted to confrontation	mid-peripheral scotoma (Goldman)	mid-peripheral constriction (no scotoma in the central 10°)	mid-peripheral constriction (no scotoma in the central 10°)	severely constricted (<10°)
Other ocular features	–	previous CME, decompensated left exophoria	previous CME	–	–	–

**Non-ocular features**						

Neurological findings	mild learning disability, spastic diplegia	mild learning disability	none reported	none reported	none reported	normal neurological examination (50 years)
Neuroimaging (age of examination)	MRI: slight hypoplasia of the splenium of the corpus callosum and the cerebellar vermis, and mild parietal increase of subarachnoid spaces (29 years)	MRI: normal (19 years)	NP	NP	NP	MRI: normal (50 years)
Other systemic features	high BMI, premature adrenarche (age 10 years)	high BMI	no	no	no	normal weight

AF, autofluorescence; BCVA, best corrected visual acuity; BMI, body mass index; CME, cystoid macula edema; ERG, electroretinogram; ERM, epiretinal membrane; EZ, ellipsoid zone; MRI, magnetic resonance imaging; NP, not performed; OCT, optical coherence tomography; OD, right eye; OS, left eye; OU, both eyes; RP, retinitis pigmentosa; -, not present.

This study adhered to the Declaration of Helsinki and was conducted in accordance with the ethical standards of the institutional and national research committees on human experimentation. Ethical approval was obtained from the following institutional review boards: Moorfields Eye Hospital and the Northwest London Research Ethics Committee (12/LO/0141); the University of California, San Diego (UCSD); the University of Washington; the UCI; the Ethikkommission Nordwest-und Zentralschweiz; and the Commission cantonale d'éthique de la recherche sur l'être humain (CER-VD). Written informed consent for participation and publication was obtained from participants (and parents where appropriate). Self-identified racial and ethnic categories were collected from all individuals as part of their standard clinical care.

**Figure 1:**
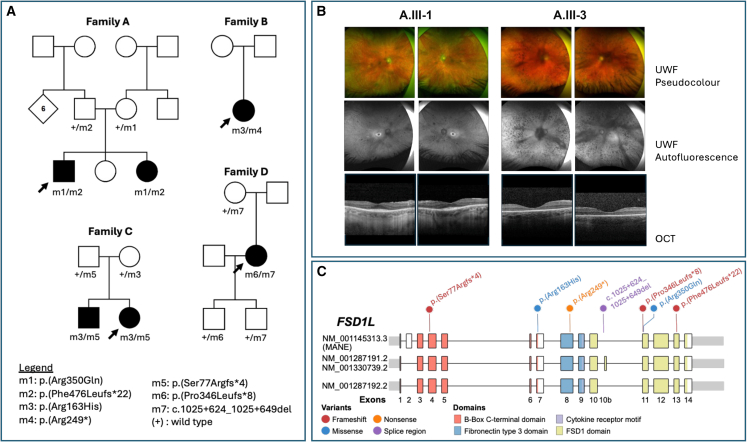
Pedigrees, representative retinal imaging, and schematic overview of *FSD1L* retinal isoforms, including the variants identified in this study (A) Pedigrees of families A–D, showing genotypes of affected individuals and segregation of *FSD1L* variants where familial data were available. The diamond symbol shows 6 additional siblings where the sex is not relevant to this study. (B) Representative multimodal retinal imaging from individuals A.III-1 (age 36 years) and A.III-3 (age 32 years), illustrating features typical of RP observed across all affected individuals. Ultra-wide-field (UWF) pseudocolor images and fundus autofluorescence (FAF) (Optos, Dunfermline, UK) show attenuated retinal vessels and mid-peripheral pigmentary changes (A.III-3), with a central ring of hyperautofluorescence demarcating surviving outer retinal structures. Macular optical coherence tomography (Spectralis OCT, Heidelberg Engineering, Heidelberg, Germany) demonstrates loss of outer retinal structures with preserved central macular structure, correlating with the FAF findings. (C) Schematic “lollipop” plot of *FSD1L* showing the location of variants identified in this study, overlaid on relevant retinal isoforms. Exons are represented as boxes and introns as horizontal lines. Protein domains and variant types are color coded. Isoforms GenBank: NM_001287191.2 and NM_001330739.2 differ only by 3 bp at the beginning of exon 11, which are also present in the canonical transcript GenBank: NM_001145313.3. The lollipop plot was drawn with ProteinPaint.

Individuals A.III-1 and A.III-3 (Figure 1A) are a brother and sister from a White British family, born to unrelated parents, with one additional unaffected sister. Both were diagnosed with RP and had features suggestive of a mild learning disability (Figure 1B). Additionally, individual A.III-1 was diagnosed with spastic diplegia in childhood, initially attributed to perinatal complications. GS was performed for individuals A.III-1 and A.III-3 and their unaffected parents (A.II-1 and A.II-2) as part of the UK 100,000 Genomes Project.^6^ Initial clinical-grade variant interrogation failed to identify a pathogenic or likely pathogenic genotype in genes listed on the PanelApp “posterior segment abnormalities” panel (encompassing 174 genes with established associations to IRDs).^8^ Subsequent research analysis focused on rare (AF < 0.001) bi-allelic protein-altering genotypes shared by both siblings and identified only a single candidate compound heterozygous genotype in *FSD1L* c.1049G>A (GenBank: NM_001145313.3) (p.Arg350Gln) and c.1428del (GenBank: NM_001145313.3) (p.Phe476Leufs^∗^22), confirmed to be inherited in *trans* (based on parental GS analysis).

Through an expanded interrogation of the Genomics England National Genomic Research Library^7^ and international collaborative efforts, four additional unrelated individuals (individuals B.II-1, C.II-1, C.II-2, and D:II-2) with non-syndromic RP and candidate bi-allelic *FSD1L* variants were identified (Figure 1A). Individual B.II-1 (Figure 1A) is a White British female and the only child of unaffected parents. Individuals C.II-1 and C.II-2 (Figure 1A) are affected siblings from a non-consanguineous White American family. Individual D.II-2 (Figure 1A) is a White Swiss female with no siblings. Across all families, there is no reported consanguinity and no known antecedents affected with a genetic eye disease. For each individual, the *FSD1L* variants identified represented the only plausible disease-associated genotype. Full details of sequencing methodology and variant prioritization for all six individuals are provided in the supplemental methods.

Five of the six affected individuals reported childhood-onset night vision difficulties (one was asymptomatic and diagnosed following identification of an affected sibling), and all exhibited an ocular phenotype consistent with rod-cone dystrophy or RP. In view of the mild neurological features described in individuals A.III-1 and A.III-3, as well as a contemporaneous report describing a severe neurological syndrome associated with bi-allelic *FSD1L* variants (in this issue of *AJHG*^9^), individual D.II-2 underwent a full neurological evaluation, including brain neuroimaging, which revealed no evidence of central nervous system involvement. Similarly, no neurological features were observed or reported in the remaining individuals. Additional clinical findings are detailed in Table 1 and Figures S1A–S1C. A total of seven *FSD1L* variants were identified, including two missense variants (c.1049G>A [p.Arg350Gln] and c.488G>A [p.Arg163His]), three frameshift variants (c.1428del [p.Phe476Leufs^∗^22], c.226_227del [p.Ser77Argfs^∗^4], and c.1037_1038delinsT [p.Pro346Leufs^∗^8]), one nonsense variant (c.745C>T [p.Arg249^∗^]), and one deep intronic deletion (c.1025+624_1025+649del [p.?]). All variants were either absent from gnomAD v.4.1.0 or observed at extremely low AFs, with no homozygous individuals identified (variant details are summarized in Figures 1C and S2A–S2C and Table 2). Segregation analysis, where available (families A, C, and D), confirmed autosomal-recessive inheritance with the two *FSD1L* alleles present in *trans* in affected individuals (Figure 1A). No bi-allelic null genotypes were observed (Figure 1A). Notably, the p.Arg163His variant was observed in two unrelated individuals, each in *trans* with a different predicted loss-of-function (pLoF) variant.

**Table 2:** *FSD1L* variants identified in this study

**Variant ID (family)**^a^	**Variant**			**Variant type**	**gnomAD AF**	***In silico* predictions**^b^					**ClinVar (ID)**	**ACMG/ACGS classification (evidence)**^c^
	**Genomic coordinates (GRCh38)**	**Nucleotide**	**Protein**			**REVEL**	**AlphaMissense**	**MutScore**	**SpliceAI**	**Pangolin**		
m1 (A)	chr9:105534516G>A	c.1049G>A	p.Arg350Gln	missense	0.00004455	0.15	0.677	0.25	0.27	0.24	absent	VUS (PM2_supp, PM3)
m2 (A)	chr9:105539309CT>C	c.1428del	p.Phe476Leufs^∗^22	frameshift	0.000001326	N/A	N/A	N/A	0.01	0.05	absent	LP (PVS1_mod, PM2_supp, PM3)
m3 (B, C)	chr9:105484404G>A	c.488G>A	p.Arg163His	missense	0.0001259	0.29	0.587	0.398	0.06	0.05	VUS (2302875)	VUS (PM2_supp, PM3_mod)
m4 (B)	chr9:105506557C>T	c.745C>T	p.Arg249^∗^	stop-gain	0.00000258	N/A	N/A	N/A	0.01	0.18	absent	VUS (PVS1_mod, PM2_supp, PM3_supp)
m5 (C)	chr9:105468210ACT>A	c.226_227del	p.Ser77Argfs^∗^4	frameshift	absent	N/A	N/A	N/A	0	0.05	absent	LP (PVS1_mod, PM3_mod, PP2_supp)
m6 (D)	chr9:105534504CA>T	c.1037_1038delinsT	p.Pro346Leufs^∗^8	frameshift	absent	N/A	N/A	N/A	0.15	N/A	absent	LP (PVS1_mod, PM3_mod, PP2_supp)
m7 (D)	chr9:105513556TT GTAAAACAGTTT CTTAACAGTTGCC>T	c.1025+624_1025+649del	p.?	intronic	0.000001337	N/A	N/A	N/A	0.07	0.27	absent	LP (PS3_mod, PM3_mod, PM2_supp)

*FSD1L* reference transcript GenBank: NM_001145313.3. AF, allele frequency; gnomAD, Genome Aggregation Database v.4.1.0; LP, likely pathogenic; N/A, not available; VUS, variant of uncertain significance.

a Variant IDs correspond to labels used in Figure 1A.

b REVEL is an ensemble score based on 13 individual scores for predicting the pathogenicity of missense variants.^10^ AlphaMissense scores can be interpreted as the approximate probability of a variant being clinically pathogenic.^11^ MutScore integrates qualitative features of DNA substitutions with new additional information derived from positional clustering.^12^ SpliceAI and Pangolin delta scores can be interpreted as the probability that the variant affects splicing at any position within a ±500 bp window around it.^13^^,^^14^ Scores for REVEL, AlphaMissense, MutScore, SpliceAI, and Pangolin range from 0 to 1, with higher scores indicating a higher probability of the variant being damaging or having a splice-altering effect.

c Variant classification and evidence codes follow American College of Medical Genetics and Genomics and Association for Molecular Pathology (ACMG/AMP) guidelines^15^ with strength modifiers (e.g., _mod and _supp) applied according to the Association for Clinical Genomic Science (ACGS) Best Practice Guidelines.^16^

**Figure 2:**
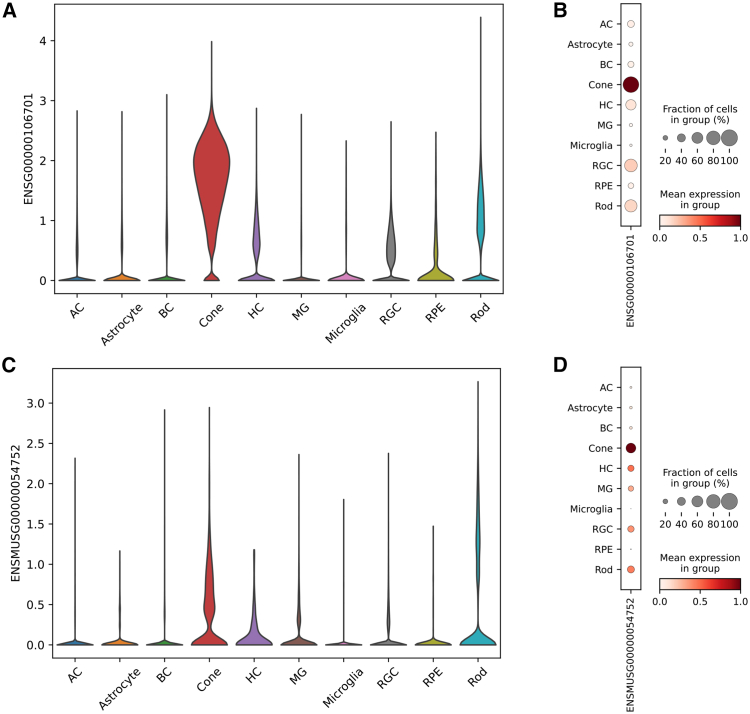
Single-cell transcriptomic profiling of *FSD1L* expression in human and mouse retinas (A and B) Violin plot (A) and dot plot (B) showing *FSD1L* expression across retinal cell types in human retina. (C and D) Violin plot (C) and dot plot (D) showing *Fsd1l* expression across retinal cell types in mouse retina. AC, amacrine cells; BC, bipolar cells; HC, horizontal cells; MG, Muller cells; RGC, retinal ganglion cells; RPE, retinal pigmented epithelium.

Consistent with the observed photoreceptor degenerative phenotype, *FSD1L* is expressed in the retina, as shown by single-cell RNA sequencing (RNA-seq) of human and mouse retina (Figure S3).^17^^,^^18^ In the human retina, *FSD1L* is most highly expressed in cone photoreceptors, with lower expression observed in rod photoreceptors, retinal ganglion cells (RGCs), and horizontal cells (HCs). Minimal to no expression is detected in amacrine cells (ACs), bipolar cells (BCs), and Müller glia (MGs) (Figures 2A and 2B). A similar expression pattern is observed in the mouse retina, with strong *Fsd1l* expression in cone cells, followed by rods, HCs, RGCs, and MGs (Figures 2C and 2D).

Within the human retina, *FSD1L* is expressed not only as the canonical (or Matched Annotation from NCBI and EBI, MANE) transcript (GenBank: NM_001145313.3) but also as at least two alternative isoforms, GenBank: NM_001330739.2 and NM_001287192.2 (Figure 1C). The non-canonical transcripts do not include exon 2, and GenBank: NM_001330739.2 includes an alternatively spliced exon, referred to here as exon 10b. This exon is conserved in mammals and shows coding constraint, supportive of a role as a functional protein-coding element.^19^ Disruption of exon 10b, therefore, may have important functional consequences, particularly in the context of the retina-enriched isoforms.

RNA-seq data^20^ obtained from human retina show that inclusion of exon 10b is above 60% in the peripheral retina (Figures S4A and S4B), with only skeletal muscle tissue having more inclusion (Figures S4C and S4D). At a cellular level, long-read single-cell sequencing of mouse retina shows exon 10b inclusion in 100% of reads from rods (128 reads) and cones (506 reads) and in 50% of reads from Müller cells (10 reads), and there is no detectable inclusion in BCs (Figure S5A). Additionally, in human macula long-read single-nuclei sequencing data, exon 10b is included in 40.05% of reads in rods (1,623 reads) and 55.19% in cones (270 reads) (Figure S5B).

One variant identified in this study, c.1025+624_1025+649del (GenBank: NM_001145313.3), is located close to the splice acceptor site of exon 10b, c.930-39_930-14del (GenBank: NM_001330739.2). *In silico* predictions suggest that both the acceptor and donor splice sites of this exon are weak (SpliceAI scores of 0.07 and 0.02, respectively), and the deletion abolishes the splice acceptor strength at the exon 10b junction (SpliceAI score of 0).^13^ The deleted region also overlaps a predicted branchpoint,^21^ representing a potential disruption of key splicing regulatory elements required for exon 10b inclusion.

**Figure 3:**
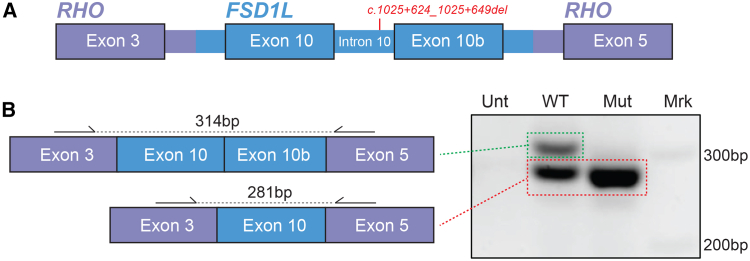
Minigene assay shows exon skipping caused by the c.1025+624_1025+649del variant (A) Schematic representation of the design of *FSD1L* pCI-NEO-RHO exon3,5/DEST minigene construct for wild type (WT) and mutant (Mut), including the variant c.1025+624_1025+649del (m7). (B) Resolution of splicing PCR products derived from ARPE-19 cells transfection with WT or Mut *FSD1L* minigenes; untransfected cells (Unt) were used as a control. Sanger sequencing confirmed the presence of the illustrated products. The filled boxes indicate exons, connected by introns. Blue color indicates the genomic fragment area cloned into the plasmid, with the vector backbone depicted in violet. Arrows indicate primer binding sites used for amplification of the cDNA. The black dotted lines refer to the amplified area on the cDNA, with their corresponding sizes. bp, base pairs; Mrk, size marker.

To investigate the impact of the *FSD1L* c.1025+624_1025+649del variant on splicing, wild-type (WT) and mutant *FSD1L* minigenes encompassing exons 10 and 10b and nearby intronic sequences were generated (Figure 3A). These minigenes were transfected into ARPE-19 cells, and splicing patterns were subsequently analyzed.

Transfection with the WT *FSD1L* minigene produced two transcripts of 314 and 281 bp. Sanger sequencing confirmed that these corresponded to transcripts with and without exon 10b, respectively (Figure 3B). As predicted, the mutant *FSD1L* minigene yielded a single 281 bp product, consistent with skipping of exon 10b; no product including exon 10b was detected. Thus, the c.1025+624_1025+649del variant leads to exon 10b skipping in ARPE-19 cells.

In tandem, we examined endogenous *FSD1L* transcripts in lymphocytes from individuals D.II-2 and D.I-1 and three controls. RT-PCR was performed on peripheral blood-derived RNA using primers spanning exons 10–14. Long-read nanopore sequencing was performed on resultant amplicons to investigate the splicing pattern, according to previous methods developed in our laboratory for low-level transcript analysis.^22^^,^^23^^,^^24^ The maximal read depth generated from individual samples was between 51,000 and 99,000. These data showed that exon 10b is included in approximately 3%–14% of *FSD1L* transcripts in the three unrelated control samples, measured as the read depth of exon 10b vs. the canonical exon 10. In contrast, it was absent in the affected individual, indicating that the intronic deletion disrupts normal splicing of this exon in lymphocytes (Figure S6). Phasing of the transcript reads from individual D.II-2 covering exon 11 showed a low proportion of reads derived from the *trans* allele carrying the c.1037_1038delinsT pLoF variant (883/5,936 reads, 15%). This suggests that transcripts derived from the pLoF allele are undergoing nonsense-mediated decay (NMD), leading to the skewed representation of the alleles. This may therefore explain why, in individual D.II-2, even though the exon 10b mis-splicing variant is heterozygous, the normally spliced exon 10b on the *trans* allele is absent due to NMD, leading to the complete absence of exon 10b in the sequencing reads.

The missense variant identified in individuals A.III-1 and A.III-3 (c.1049G>A [p.Arg350Gln]) affects a residue on an unstructured loop in the predicted 3D model (Figure S2C) but also had a SpliceAI high recall score (Δ0.27, Table 2). Examination of endogenous transcripts in lymphocytes from A.III-3, compared to her unaffected father (A.II-1) carrying c.1428del (p.Phe476Leufs^∗^22), showed a low level of skipping of exon 11 (8.9% of canonical transcripts, Figure S7), which would lead to an out-of-frame truncation terminating in exon 12. These data suggest that the effect of this allele may be a combination of LoF and the amino acid substitution.

Together, these findings suggest a weakening of splicing of exon 10b consequent upon the intronic deletion and specifically affecting transcript GenBank: NM_001330739.2, which may represent a retina-enriched isoform. Of note, given the identification of a severe neurodevelopmental phenotype association with bi-allelic pLoF and severe variants in this gene (in this issue of *AJHG*^9^), disrupted splicing of this retina-enriched exon may represent a mechanism for the sparing of non-retinal neurons. For other families, subtle neurological features may not yet be evident, given the young age of some affected individuals, and longitudinal follow-up will be important to determine whether additional manifestations emerge with time.

*FSD1L*, located on chromosome 9q31.2, was originally identified based on its sequence homology to *FSD1* (MIM: 609828), a gene on chromosome 19q13.33 that shows preferential expression in the brain. *FSD1L* encodes a cytoplasmic protein that closely resembles its paralog FSD1, sharing approximately 50% amino acid identity and 65%–70% similarity across the full-length protein.^25^ Both FSD1L and FSD1 contain an N-terminal coiled-coil domain, a central fibronectin type III (FN3) motif, and a C-terminal SPRY domain—highly conserved elements implicated in protein-protein interactions.^25^

Although FSD1L has not been functionally characterized in detail, its homolog FSD1 (also known as MIR1) has been reported to bind and stabilize microtubules, potentially via its C-terminal SPRY domain; it is also described as exhibiting dynamic localization throughout the cell cycle—associating with centrosomes in interphase and redistributing during mitosis—consistent with a role for FSD1 in centrosomal positioning and cytoskeletal regulation.^26^ Given the high degree of sequence similarity and conserved domain architecture, it is plausible that FSD1L may exhibit similar properties. Indeed, both FSD1 and FSD1L exhibit similarity to ubiquitin ligases of the TRIM (Ring-B-box-coiled-coil, RBCC) family.^27^ In addition, FSD1L shows preferential expression in ciliated cells and localizes to ciliary structures in human bronchus and fallopian tubes.^28^

**Figure 4:**
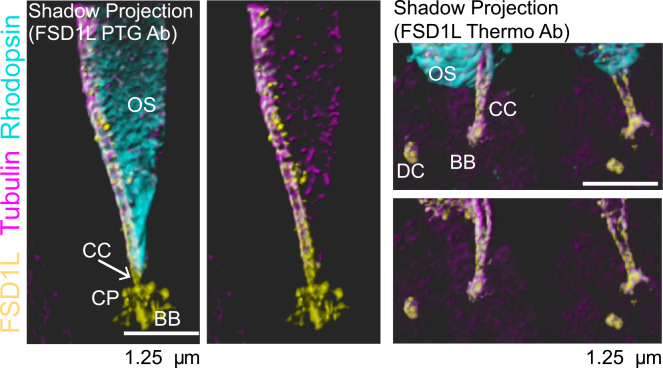
FSD1L localizes to the photoreceptor cilium in human retina 3D shadow projections of confocal z stacks from human rod photoreceptors labeled with tubulin (magenta), rhodopsin (cyan), and FSD1L (yellow) after expansion.^29^ BB, basal body; CC, connecting cilium; CP, ciliary pocket; DC, daughter centriole; OS, outer segment. Scale bars: 1.25 μm corrected for 4× expansion.

To gain insight into the localization of the FDS1L protein in the human retina, we performed immunofluorescence staining and ultrastructure expansion microscopy (U-ExM). These techniques, using antibodies that detect most retinal isoforms of FSD1L (including that encoded by GenBank: NM_001330739.2 containing exon 10b), revealed localization of FSD1L along the photoreceptor microtubule axoneme (Figures 4 and S8A–S8C). Using rhodopsin as the outer segment marker, FSD1L was observed to colocalize with tubulin from the basal body throughout the length of the connecting cilium and into the outer segment axoneme. Localization was also observed in what appears to be the ciliary pocket, an inner segment region surrounding the connecting cilium, using one of the two antibodies specific for FSD1L.

To our knowledge, variants in *FSD1L* have not previously been implicated in heritable disease. In this study, we identify bi-allelic *FSD1L* variants in four unrelated families with RP. We show that FSD1L is expressed in the retina, particularly in photoreceptors, and that the protein localizes to the photoreceptor microtubule axoneme, including the connecting cilium and outer segment. Furthermore, we show that a deep intronic variant identified in an individual with non-syndromic RP disrupts splicing of a conserved, retina-enriched exon (exon 10b), supporting a tissue-specific isoform effect as a potential mechanism for isolated retinal disease in this individual. In summary, these findings provide evidence supporting bi-allelic disruption of *FSD1L* as a mechanism of IRD. Notably, individuals A.III-1 and A.III-3 are affected with both retinal dystrophy and a mild neurodevelopmental disorder, supporting a role for *FSD1L* in both retinal and neurological function.

*FSD1L*-associated disease may therefore span a broad phenotypic spectrum, ranging from severe neurodevelopmental syndromes to, at its mildest, non-syndromic retinal dystrophy, where the specific combination and functional severity of the two alleles likely determines the clinical outcome. Here, the phenotype observed in affected individuals in families A–C is likely to be driven by non-LoF alleles and may be expected to have a milder effect on the protein. Affected individuals B.II-1, C.II-1, and C.II-2, who harbor a missense variant with a pLoF allele, likely retain partial FSD1L function, resulting in a retinal phenotype. Individuals A.III-1 and A.III-3 harboring a pLoF allele in *trans* with a missense variant shown to cause low-level exon skipping and out-of-frame truncation exhibit the retinal phenotype with an associated mild neurological presentation. A subtle or late-onset neurological component in individuals B.II-1, C.II-1, and C.II-2 cannot be excluded, particularly as formal neuroimaging could not be obtained, and some affected individuals are still in early adolescence. For D.II-2, the intronic deletion specifically disrupting the retina-enriched exon 10b provides a plausible mechanism for the absence of neurological findings.

Taken together, these observations highlight the importance of considering isoform- and allele-specific effects in the interpretation of *FSD1L* variants. We recommend that disruption of both the canonical (GenBank: NM_001145313.3) and retina-enriched (GenBank: NM_001330739.2) isoforms be considered in diagnostic variant assessment, given the full spectrum of *FSD1L*-associated disease—from isolated retinal dystrophy to syndromic neurodevelopmental presentations.

While our findings identify a role for FSD1L disruption in IRDs, the specific molecular mechanisms leading to retinal degeneration remain to be elucidated. Given the reliance of photoreceptors on microtubule-based trafficking for the delivery of proteins to the outer segment and maintenance of cell polarity,^30^ disruption of this process through FSD1L dysfunction provides a plausible mechanism for the retinal degeneration observed in affected individuals with bi-allelic *FSD1L* variants. Functional studies in additional model systems may further clarify the role of *FSD1L* in retinal health and inform how disruption of specific isoforms or protein domains contributes to retinal disease pathogenesis.

## Data and code availability

• Affected individual data supporting the findings of this study are confidential and subject to ethical restrictions. Access to data can be requested from the corresponding author, subject to appropriate approvals and compliance with data protection regulations.
• Research on the de-identified individual data used in this publication can be carried out in the Genomics England Research Environment, subject to a collaborative agreement that adheres to individual-led governance. All interested readers will be able to access the data in the same manner as the authors did. For more information about accessing the data, interested readers may contact research-network@genomicsengland.co.uk or access the relevant information on the Genomics England website: https://www.genomicsengland.co.uk/research.

## Acknowledgments

The authors thank the individuals and their families for their participation in this study. This study was supported by the 10.13039/501100000265Medical Research Council (Clinician Scientist Fellowship, grant reference UKRI440 [S.L.]), Fight for Sight UK (Early Career Investigator Award, grant no. 5045/46 [G.A.]), 10.13039/100000002National Institutes of Health (NIH; grant nos. NIH-P20GM139769 [G.A.], EY022356 [R.C.], and EY018571 [R.C.]), the Retinal Research Foundation (R.C.), the Wellcome Trust (grant no. 206619/Z/17/Z [O.A.M.]), the 10.13039/501100000272National Institute for Health and Care Research (NIHR) Manchester Biomedical Research Centre (BRC) (NIHR203308), and the NIHR-BRC at Moorfields Eye Hospital and the UCL Institute of Ophthalmology. C.R. was supported by the 10.13039/501100001711Swiss National Science Foundation (SNSF) grant no. 310030_204285. M.Q. was supported by the RetinAward 2021. The authors acknowledge support to the Gavin Herbert Eye Institute at the University of California, Irvine, from an unrestricted grant from Research to Prevent Blindness and from NIH core grant P30 EY034070. We thank the Imaging Core Facility (IMCF, Biozentrum, University of Basel) for use of the Stellaris 8 Falcon microscope. We also thank Dr. Anna Pichiecchio for her assistance in preparing the neuroimaging figures during the early stages of this work. This research was made possible through access to data and findings in the National Genomic Research Library; please see the supplemental information for the full acknowledgments. The funding organizations had no role in the design or conduct of this research. The views expressed are those of the authors and do not necessarily represent those of the funding organizations, NHS, NIHR, or the Department of Health.

## Author contributions

Conceptualization (ideas, formulation, or evolution of overarching research goals and aims), C.R., R.C., and G.A.; formal analysis (application of statistical, mathematical, computational, or other formal techniques to analyze or synthesize study data), Y.C., A.R.M., A.B.I.R., M.Q., M.W., and S.L.; investigation (conducting a research and investigation process, specifically performing the experiments, or data/evidence collection), S.L., F.C., Y.C., A.J.L., A.R.M., V.V., F.P., A.B.P., E.R.S., M.U., A.B.I.R., K.K., A.J., M.F., S.W., M.M.R., V.H., S.P., D.M., A.T., R.S., Y.L., J.L., M.W., O.A.M., S.B., M.Q., K.P., A.R.W., C.R., R.C., and G.A.; resources (provision of study materials, reagents, materials, patients, laboratory samples, animals, instrumentation, computing resources, or other analysis tools), M.F. and S.W.; writing – original draft (preparation, creation, and/or presentation of the published work, specifically writing the initial draft [including substantive translation]), S.L., F.C., Y.C., M.Q., A.R.M., C.R., R.C., and G.A.; writing – review & editing (preparation, creation, and/or presentation of the published work by those from the original research group, specifically critical review, commentary, or revision, including pre- or post-publication stages), S.L., F.C., Y.C., A.J.L., A.R.M., V.V., F.P., A.B.P., E.R.S., M.U., A.B.I.R., K.K., A.J., M.F., S.W., M.M.R., V.H., S.P., D.M., A.T., R.S., Y.L., J.L., M.W., O.A.M., S.B., M.Q., K.P., A.R.W., C.R., R.C., and G.A.; visualization (preparation, creation, and/or presentation of the published work, specifically visualization/data presentation), S.L., Y.C., A.R.M., M.Q., F.C., A.B.I.R., C.R., R.C., and G.A.; supervision (oversight and leadership responsibility for the research activity planning and execution, including mentorship external to the core team), C.R., R.C., and G.A.; funding acquisition (acquisition of the financial support for the project leading to this publication), C.R., R.C., and G.A.

## Declaration of interests

The authors declare no competing interests.

## Web resources

GenBank, https://www.ncbi.nlm.nih.gov/genbank/
OMIM, https://www.omim.org

## Supplemental information

Document S1. Figures S1–S8, supplemental notes, and supplemental methods

Document S2. Article plus supplemental information
