# Supplementary material for: Bi-allelic variants in FSD1L cause retinitis pigmentosa with or without neurological involvement
Source: Am J Hum Genet. 2026 Feb 19;113(3):616–26. doi: 10.1016/j.ajhg.2026.01.015 (PMC13087403; doi:10.1016/j.ajhg.2026.01.015)
Supplement: Document S1. Figures S1–S8, supplemental notes, and supplemental methods [file mmc1.pdf]

The American Journal of Human Genetics, Volume *113*

Supplemental information

Bi-allelic variants in *FSD1L* cause retinitis pigmentosa with or without neurological involvement

Siying Lin, Francesca Cancellieri, Yexuan Cao, Andrew J. Lotery, Abigail R. Moye, Veronika Vaclavik, Fabienne Perren, Andrzej B. Poplawski, Elena R. Schiff, Mukhtar Ullah, Ana Belen Iglesias-Romero, Karolina Kaminska, Aleksandr Jestin, Marc Folcher, Sandrine Wallerich, Mariana M. Ribeiro, Vincent Hahaut, Simone Picelli, Debarshi Mustafi, Aleksander Tworak, Roman Smidak, Yumei Li, Jiaxiong Lu, Meng Wang, Omar A. Mahroo, Shyamanga Borooah, Mathieu Quinodoz, Krzysztof Palczewski, Andrew R. Webster, Carlo Rivolta, Rui Chen, and Gavin Arno

SUPPLEMENTAL INFORMATION

Clinical findings

Individual A.III-1 is a White British male with one affected sister, was first noted to have poor night vision at the age of 9 and was subsequently diagnosed with retinitis pigmentosa (RP) at age 10.

He was born following an uneventful pregnancy and labor induced at 42 weeks' gestation, and was delivered breech via the vaginal route. He began walking at 11 months but was noted to walk on his toes, with an unsteady, wobbling gait. This led to a diagnosis of mild cerebral palsy with spastic diplegia, initially attributed to the difficult delivery at birth. He has a mild learning disability but progressed through mainstream junior and high schools and later attended college. His medical history includes a diagnosis of premature adrenarche at age 10, although urinary steroid profiling excluded significant adrenal dysfunction. He was also noted to have a high body mass index (BMI). An MRI brain scan at age 29 showed slight hypoplasia of the splenium of the corpus callosum and the cerebellar vermis, as well as mild parietal widening of the subarachnoid spaces.

At his most recent eye examination aged 36, his Snellen best corrected visual acuity (BCVA) was 20/40 in the right eye and 6/50 in the left eye. His visual fields to confrontation were severely constricted. He had mild myopic astigmatism, with a refraction of +0.50/-3.50 x 11° in the right eye and +0.25/-3.50 x 176° in the left. Anterior segment examination was unremarkable with clear media and normal intraocular pressures. Fundus examination revealed a hypopigmented fundus with prominent choroidal vasculature and bilateral atrophic changes at the macula. There was no evidence of pigmentary retinopathy. The optic discs were pale in appearance with attenuated retinal vessels noted. Autofluorescence imaging showed a small hyperautofluorescent ring. Optical coherence tomography (OCT) scans showed a mild epiretinal membrane in both eyes and loss of the perifoveal ellipsoid zone with subfoveal sparing, and no cystoid macular edema (CME) in either eye.

Electroretinography (ERG) testing performed at ages 11 and 19 years showed generalized retinal dysfunction affecting both rod and cone systems. Initially, macular function was spared; however, the follow-up examination demonstrated a progression to moderately severe rod-cone dystrophy with marked macular involvement bilaterally, with evidence of deterioration in both macular and generalized retinal function.

Individual A.III-3 is the affected younger sister of Individual A.III-1, who was noted to have poor night vision in childhood, and was diagnosed with RP aged 13, following her brother's diagnosis

She was born 7 days post-term via emergency caesarean section for breech presentation, after an otherwise uncomplicated pregnancy. She achieved normal developmental milestones and began walking at around 11 months. However, she had a long-standing history of clumsiness, leading to a suspected diagnosis of dyspraxia or ataxia. MRI brain imaging at age 19 was reported as normal. Given the combination of

neurological and retinal features, a muscle biopsy was performed to evaluate for mitochondrial cytopathy, which was subsequently ruled out. She has a mild learning disability, completed mainstream schooling and college, and was also noted to have a high BMI.

Her ocular history includes decompensated left exophoria, surgically corrected at age 30, and prior bilateral CME that had been poorly responsive to topical and oral carbonic anhydrase inhibitors but later resolved.

At her last eye examination at age 32 years, BCVA was 20/120 in the right eye and 20/200 in the left eye. Visual fields to confrontation were severely constricted. She had mild myopic astigmatism, with a refraction of +0.75/-2.75 x 16° in the right eye and +1.00/-5.75 x 147° in the left eye. Anterior segment examination revealed bilateral posterior subcapsular opacities, worse on the left compared to the right, with intraocular pressures within normal limits. Fundus examination showed a hypopigmented fundus with prominent choroidal vasculature, optic disc pallor, attenuated retinal vessels, and peripheral pigmentary retinopathy. Autofluorescence imaging showed mid-peripheral hypoautofluorescence with a central ring of hyperautofluorescence. OCT scans showed loss of the perifoveal ellipsoid zone with subfoveal sparing, and no CME in either eye (Figure 1)

ERG testing at age 15 showed a generalized retinal dysfunction affecting rod and cone photoreceptors, consistent with a moderately severe rod cone dystrophy with marked macular involvement bilaterally.

Individual B.II-1 is a White British female, who presented at age 5 years with nyctalopia and was subsequently diagnosed with retinitis pigmentosa (RP). She underwent bilateral cataract surgery (right eye at age 30, left eye at age 27) and is pseudophakic in both eyes. She has a history of CME, which has since resolved, and mid-peripheral visual field loss documented on Goldmann perimetry. At her last examination at age 32 years, BCVA was 20/50 in the right eye and 6/80 in the left eye. Fundus examination showed pale optic discs, attenuated retinal vessels and mid peripheral pigmentary retinopathy. There was widespread hypoautofluorescence with a small central area macular hyperautofluorescence noted on autofluorescence imaging. ERG testing at age 16 years showed severely reduced rod and cone responses. There were no neurological features reported or observed at her most recent follow-up visit.

Individual C.II-1 is a White American female with one affected brother. She presented at age 14 with nyctalopia and was subsequently diagnosed with RP. She underwent bilateral cataract extraction with intraocular lens implantation, and was pseudophakic in both eyes at the time of her last evaluation, also at age 14.

Snellen BCVA was 20/50 in both eyes. Fundus examination showed bone spicule pigmentation in the superonasal region, normal-appearing optic discs, and bilateral macular oedema with loss of the foveal reflex. Fundus autofluorescence imaging showed peripheral hypoautofluorescence without a central hyperautofluorescent ring. OCT scans demonstrated CME, disruption of the parafoveal ellipsoid zone, and preservation

of the posterior vitreous. Visual field testing using microperimetry indicated markedly reduced central retinal sensitivity, although no absolute scotoma was observed within the central 10 degrees. At this time, there were no reported or observed neurological or systemic abnormalities.

Individual C.II-2 is the younger brother of C.II-1, who is similarly affected. He is a White American male who was asymptomatic at the time of his last evaluation at age 12, and was diagnosed with non-syndromic RP following ophthalmic assessment prompted by his sister's diagnosis, with no evidence of neurological involvement. He was phakic with clear lenses in both eyes.

Snellen BCVA was 20/32 in both eyes. Fundus examination revealed loss of the foveal reflex with bilateral macular edema but was otherwise unremarkable. Fundus autofluorescence imaging showed peripheral hypoautofluorescence without central hyperautofluorescence. OCT imaging demonstrated loss of the perifoveal ellipsoid zone with preservation of the subfoveal region, along with CME. Visual field testing revealed mid-peripheral constriction, with central 10-degree sensitivity retained. ERG and neuroimaging were not available. No neurological or systemic abnormalities were noted at the time of examination.

Individual D.II-2 is a White Swiss female, who presented at age 10 with nyctalopia and high myopia. At her most recent evaluation at age 52, she was pseudophakic in both eyes following bilateral cataract surgery at age 36. BCVA was 20/1200 in the right eye and 20/400 in the left eye. Fundus examination revealed a hypopigmented posterior pole with macular atrophy, pale optic discs, attenuated retinal vessels, and widespread pigmentary retinopathy. Fundus autofluorescence imaging showed hypoautofluorescence throughout the mid-periphery and posterior pole, with a residual area of preserved autofluorescence within the vascular arcades. OCT scans demonstrated retinal thinning and loss of the ellipsoid zone, with no evidence of CME. ERG testing performed at age 42 showed undetectable scotopic and photopic responses. Visual field testing revealed severe constriction, with residual fields reduced to less than 10 degrees.

She underwent a full neurological evaluation, including MRI brain imaging at age 50, which did not show any evidence of central nervous system involvement.

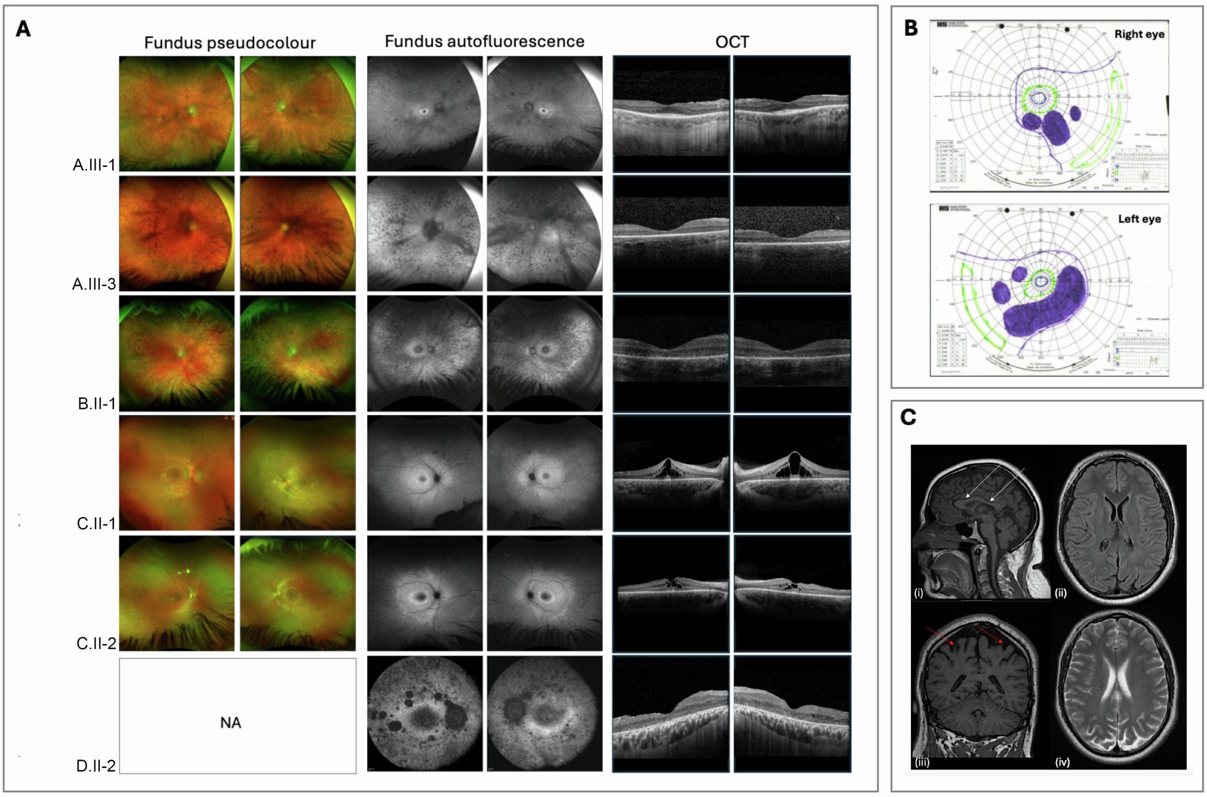

Supplemental Figure S1: Clinical findings in individuals with biallelic *FSD1L* variants. A: Multimodal retinal imaging, including ultra-widefield pseudocolour (Optos plc, Dunfermline, UK), fundus autofluorescence images (Optos ultra-widefield and Heidelberg Spectralis, Heidelberg Engineering, Heidelberg, Germany), and macular optical coherence tomography (OCT) scans (Heidelberg Spectralis and Topcon Corporation, Tokyo, Japan)]. Findings across all individuals are consistent with a rod-cone retinal dystrophy or retinitis pigmentosa (RP). Of note, macula edema appears to be a common associated feature, present at the most recent evaluation in Individuals C.II-1 and C.II-2, and previously documented in Individuals A.III-3 and B.II-1. **B**: Goldmann visual field testing from Individual B.II-1, showing a mid-peripheral scotoma in both eyes with preserved central fields. **C**: Brain MRI of Individual A.III-1. T1-weighted sagittal and axial images (i, iii), axial FLAIR (ii), and T2-weighted axial images (iv) show slight thinning of the corpus callosum (white arrows, [i]) with no evidence of white matter signal abnormality (ii, iv). Mild parietal atrophy was also noted (red arrows, [iii]).

***In silico* structural modelling and interpretation of selected *FSD1L* truncating and missense variants**

The p.(Arg249*), p.(Ser77Argfs*4) and p.(Pro346Leufs*8) variants are all predicted to undergo nonsense-mediated decay (NMD), consistent with a LOF mechanism. In contrast, the c.1428del; p.(Phe476Leufs*22) variant introduces a frameshift in the penultimate exon that leads to a premature stop codon in the final exon. This frameshift falls within a region that may allow transcripts to escape NMD[1,2], potentially allowing translation of a truncated protein with an altered C-terminus. Structural modelling of the p.(Phe476Leufs*22) variant (Supplemental Figure 6A) shows loss of the final β-strand and terminal α-helix of the SPRY domain, features that are present in the wild-type structure. This C-terminal truncation likely compromises the overall structural integrity of the SPRY fold, with potential consequences for protein stability or disruption of protein–protein interactions. The C-terminal SPRY domain of FSD1 has been shown to mediate microtubule binding[3], raising the possibility that disruption of the homologous region in

FSD1L may similarly impair intracellular trafficking pathways critical for photoreceptor maintenance and function.

To assess the impact of the two identified missense variants, p.(Arg163His) and p.(Arg350Gln), structural models were generated and compared with the wild-type protein (Supplemental Figure S2B and S2C). Arg163 is located at the C-terminal boundary of the coiled-coil domain and projects into a tightly packed helical region. Substitution of arginine with histidine introduces a shorter, less positively charged side chain, which may disrupt local packing, potentially affecting protein stability or oligomerisation. In contrast, Arg350 lies within a flexible, exposed loop linking two folded domains. The Arg350Gln substitution alters the local surface charge by replacing a long, positively charged side chain with a polar but uncharged glutamine, potentially also disrupting loop conformation or transient protein-protein interactions. Alternatively, the p.(Arg350Gln) variant may exert its effect through disrupted splicing, with a possible out-of-frame skipping of exon 11, as suggested by *in silico* predictions (Main manuscript; Table 2).

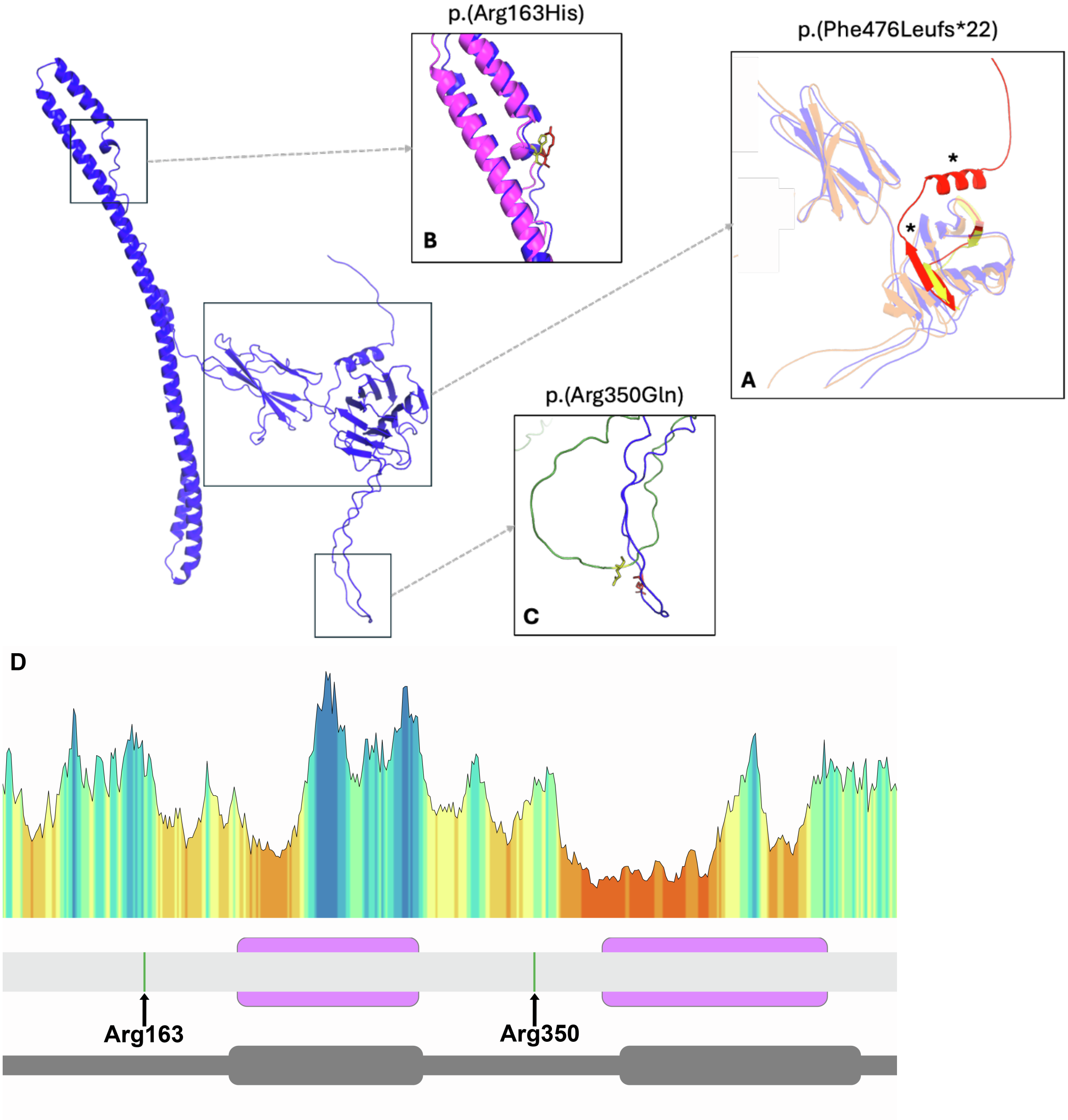

Supplemental Figure S2. Structural 3D modelling of wild-type FSD1L and selected variants identified in this study. The full-length structure of wild-type (WT) FSD1L is shown in blue (left). **A**: Overlay of the WT (blue) and truncated frameshift mutant (orange) structures, showing the overlapping C-terminal region beginning at residue 476. In this view, the WT segment from residue 476 onward is shown in red, while the corresponding truncated region of the mutant protein is shown in yellow. The p.(Phe476Leufs22) frameshift variant, if translated, leads to premature termination, resulting in loss of the final β-strand and α-helix of the SPRY domain (*). **B**: Zoomed-in view of residue 163. The magenta cartoon represents the Arg163His mutant structure overlaid on WT. **C**: Zoomed-in view of residue 350. The dark green cartoon represents the Arg350Gln mutant structure overlaid on WT. In both panels (A) and (B), WT side chains are shown in red sticks and corresponding mutant side chains in yellow sticks. **D:** Metadome 2.0 mutation tolerance visualization for FSD1L NM_001145313.1 with Arg163 and Arg350 positions highlighted showing the relative tolerance at these regions. All structural models were generated using AlphaFold (via ColabFold, v1.5.5) and visualised in PyMOL (v3.1.0)[4,5].

Cell type-specific expression of *FSD1L* and *FSD1* in human and mouse retinas

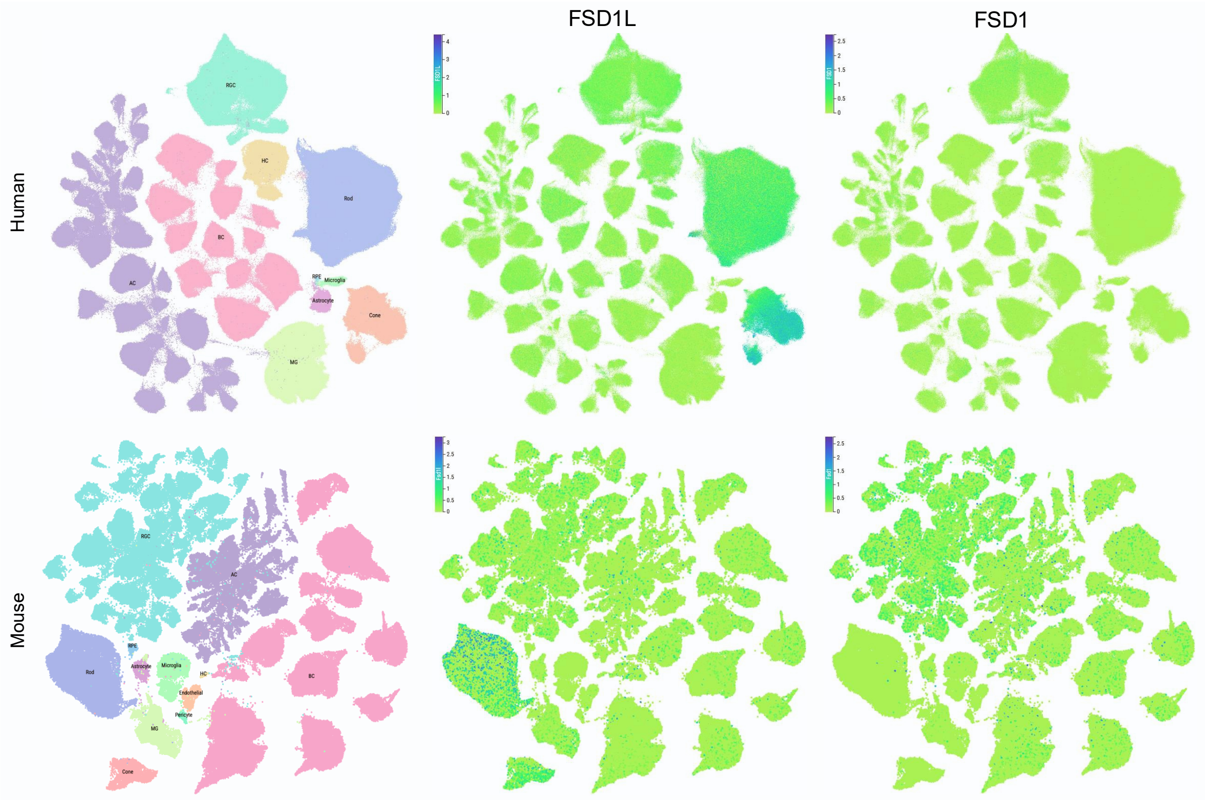

Supplemental Figure S3: Single-cell transcriptomics of *FSD1L* and *FSD1* gene expression pattern in human and mouse retina. (Top panel) Expression levels of *FSD1L* and *FSD1* across retinal single-cell clusters in the human retina. (Bottom panel) Expression levels of *FSD1L* and *FSD1* across retinal single-cell clusters in the mouse retina.

Differential *FSD1L* exon usage across regions of the human retina and other tissues

To assess how *FSD1L* exon usage varies across the retina and other tissues, we analyzed the inclusion of two exons – exon 2 and the alternatively spliced exon 10b - using RNA-seq data. In the human retina, RNA expression analysis[6] show that exon 2 is largely excluded across all regions of the retina (Supplemental Figure S4A). In contrast, exon 10b showed marked regional variability, with the highest inclusion in the peripheral retina and the lowest in the central retina (Supplemental Figure S4B). These findings highlight region-specific alternative splicing events that may reflect differential isoform usage across retinal compartments, likely with a higher exon 10b retention in rods, which are much more frequent in the peripheral retina.

To evaluate expression of the same two exons more broadly across different human tissues, we analyzed RNA-seq data from GTEx (V10)[7]. This revealed that exon 2 was broadly excluded across most systemic tissues (Supplemental Figure S4C), while exon 10b showed the highest expression in the muscle and retina (Supplemental Figure S4D). These data support the hypothesis that exon 10b is an alternatively spliced exon of two isoforms enriched in the retina.

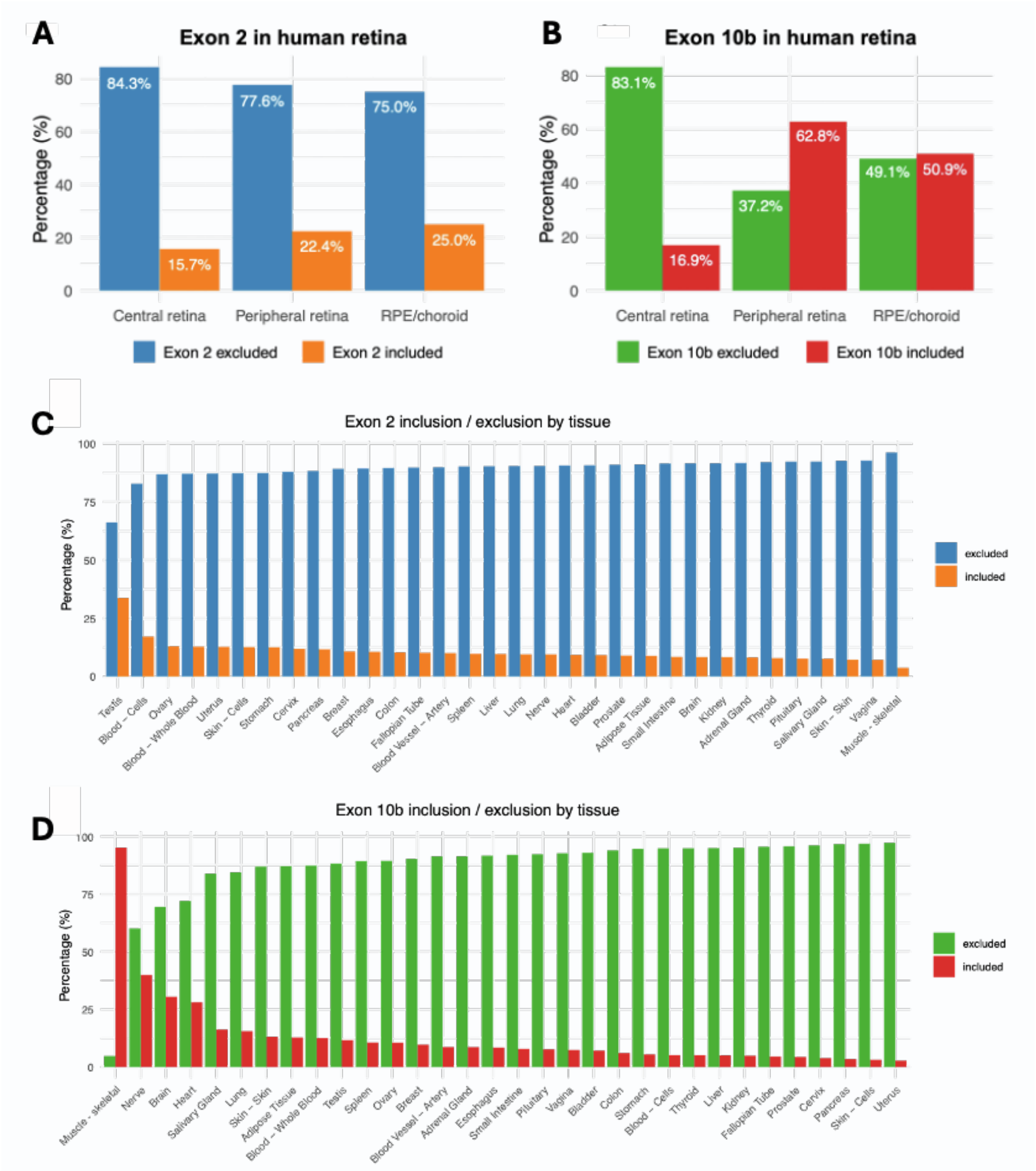

Supplemental Figure S4: A,B: Proportion of *FSD1L* transcripts including or excluding exon 2 (A) and exon 10b (B) across three retinal compartments: central retina, peripheral retina, and RPE/choroid. **C,D:** Proportion of exon 2 (C) and exon 10b (D) inclusion or exclusion across human tissues from GTEx (V10). Data are plotted as percentages, with included and excluded isoforms colour-coded accordingly.

Single-cell long-read RNA-seq of mouse and human retina

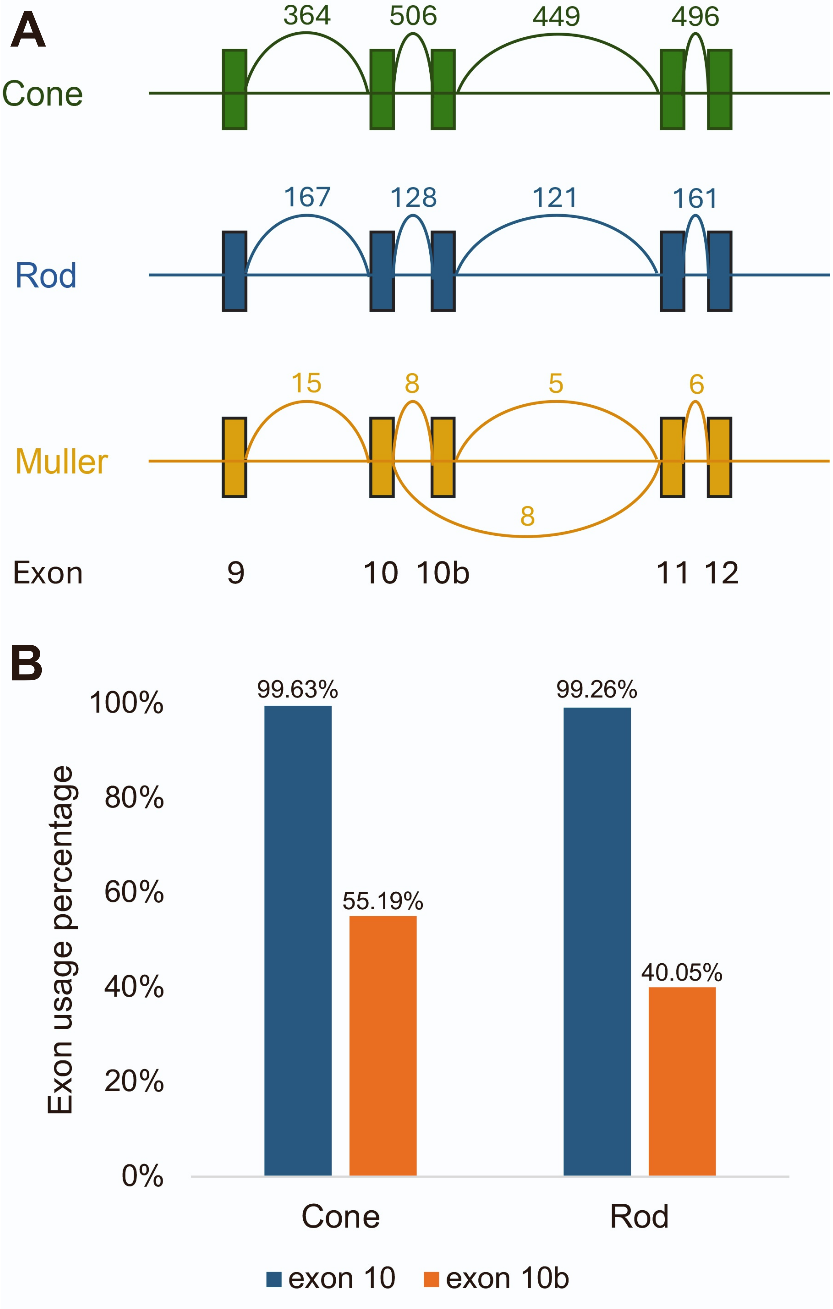

Supplemental Figure S5 *FSD1L* exon 10b usage in cone and rod photoreceptors based on long-read snRNA-seq in mouse peripheral retina **(A)** and human macula tissue **(B)**.

FSD1L RT-PCR nanopore amplicon sequencing

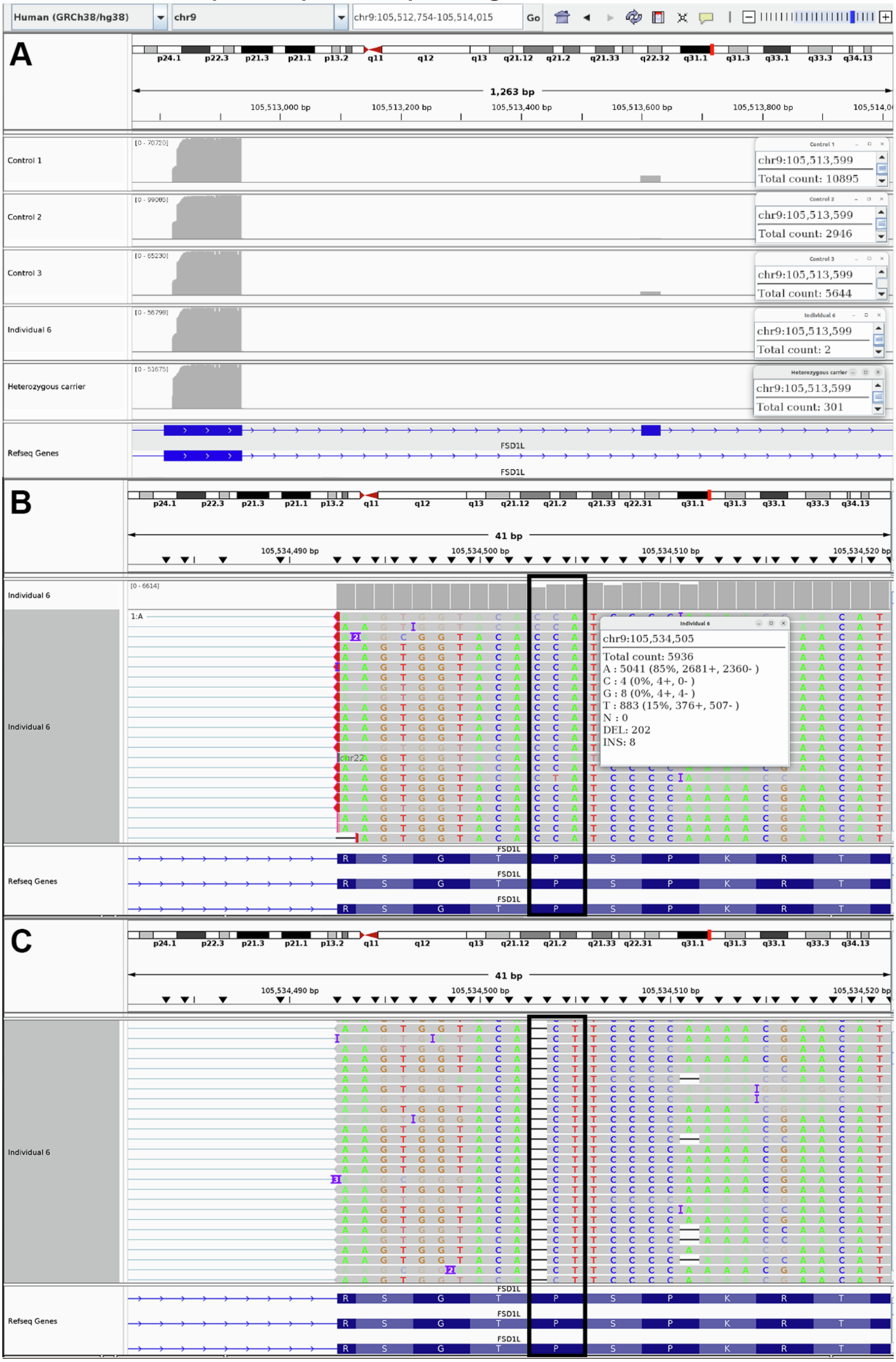

Supplemental figure S6: Integrative Genome Viewer (IGV) images representing data from *FSD1L* RT-PCR nanopore amplicon sequencing experiments. A: The relative read-depth for exon 10 and 10b from control 1-3 (unrelated donor samples), Individual D.II-2 and D.I-1. Overall read-depth (exon 10) ranged between approximately 51,000-99,000 with exon 10b covered in approximately 3-14% of reads in controls. Exon 10b coverage was 0.6% in the carrier and 0 in individual D.II-2. **B and C:** Coverage of the 5' of exon 11 for D.II-2 split by allele (B: wildtype at this position corresponding to the exon 10b splice variant allele, C: the c.1037_1038delinsT allele). The indel position is highlighted by the black rectangle. Highlighted in the text box is the allele distribution at the indel (c.1037_1038delinsT) represented by the nucleotide call at position c.1038, corresponding to the third base of the proline codon (p.Pro346). The A call is wildtype and the T call here corresponds to the indel allele (reads in panel C) and is only observed on 15% of reads, indicating a skew, likely due to nonsense-mediated decay of the frameshifting indel allele.

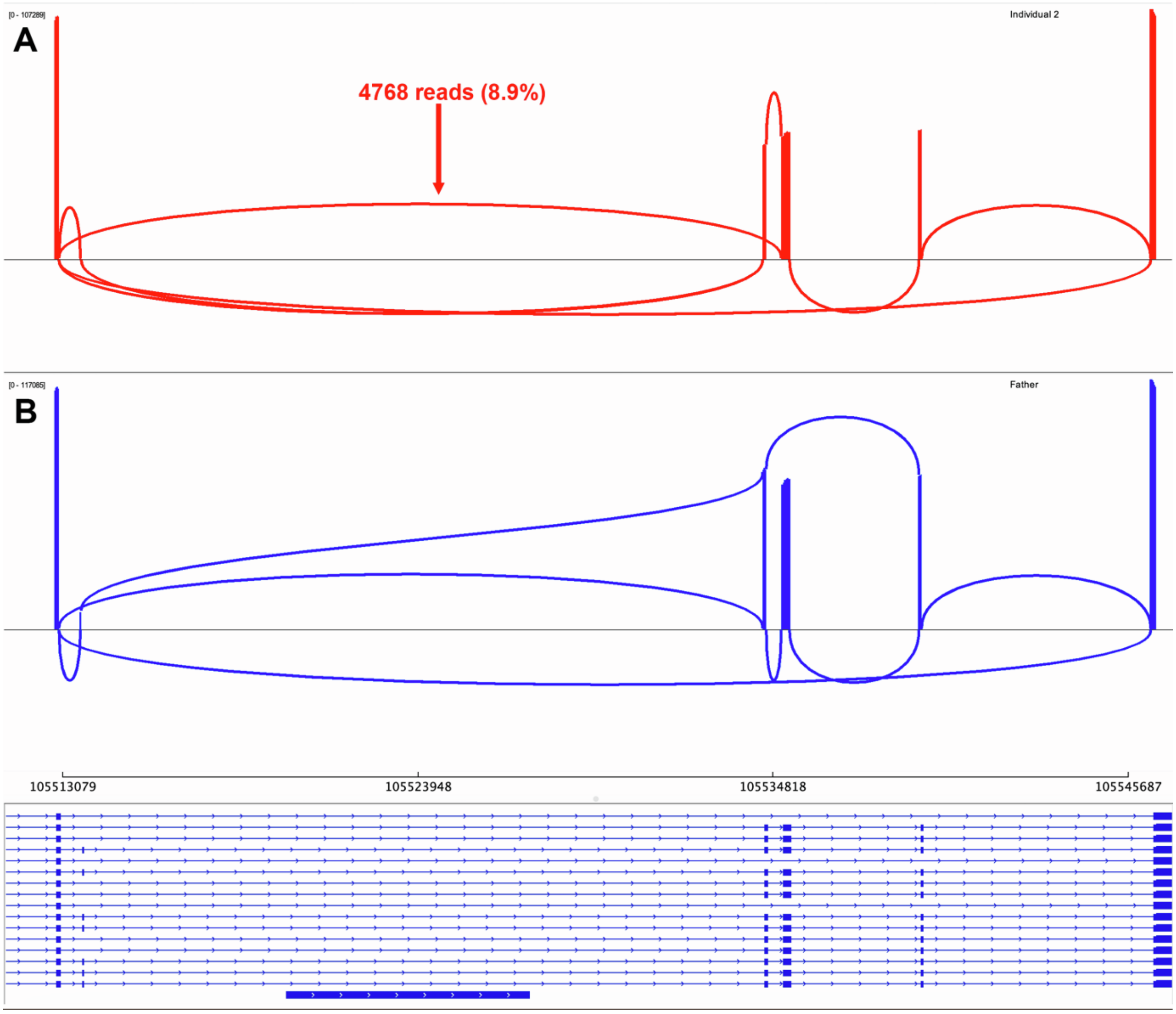

Supplemental figure S7: IGV sashimi plots representing RT-PCR nanopore amplicon sequencing experiments. A: Amplification of *FSD1L* exons 10-14 from Individual A.III-3 blood derived mRNA highlighting the novel splice junction between exon 10 and exon 12 (exon 11 skipping) observed in 4768 reads (8.9%) of amplicons derived from the canonical transcript (NM_001145313.3, 53,488 reads). **B:** Amplification of *FSD1L* exons 10-14 from A.II-1 harboring the heterozygous c.1428del; p.(Phe476Leufs*22) allele showing zero reads with exon 10/12 junctions. The Refseq gene track is shown underneath.

Expansion microscopy confirms FSD1L localization to the photoreceptor cilium in human retina

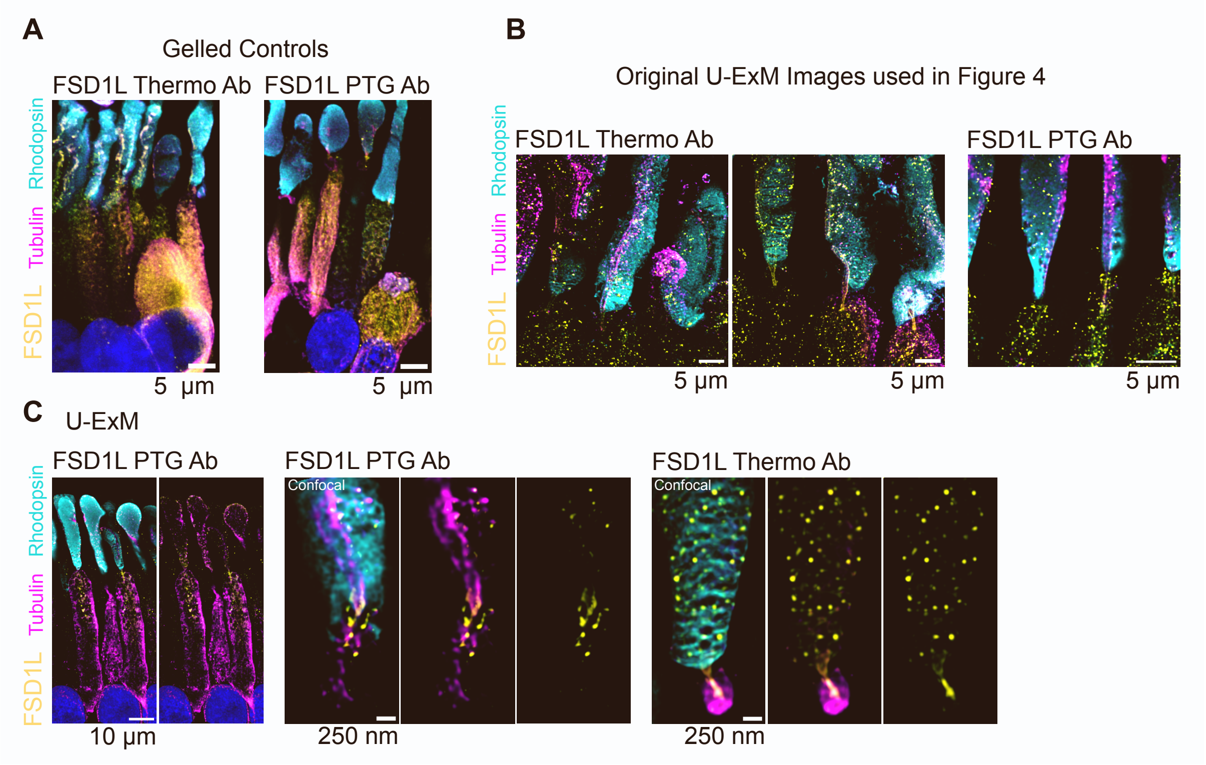

Supplemental Figure S8: FSD1L localization to the photoreceptor cilium revealed by expansion microscopy. A: Gelled controls of human retina tissue before undergoing expansion. **B:** Original max projections of confocal z-stack images used in Figure 4. **C:** Max projections of confocal z-stack images from human rod photoreceptors labelled with tubulin (magenta), rhodopsin (cyan), and FSD1L (yellow) after expansion[8]. Scale bars: 5µm, 10µm, 250nm.

Supplemental methods

Genetic testing, variant annotation and prioritization

Clinical-grade sequencing and initial variant analysis

Genome sequencing (GS) for Family A (Individuals A.II-1, A.II-2, A.III-1, A.III-3) was performed as part of the UK 100,000 Genomes Project (100kGP), and for B.II-1 through the NHS Genomic Medicine Service (GMS), with clinical pipeline analysis focusing on protein-altering variants, including single nucleotide variants (SNVs), short insertion-deletion variants (INDELs) and structural variants (SVs) and copy number variants (CNVs), using Starling (Isaac Variant Caller)[9], Manta[10], Canvas[11] (100kGP) and DRAGEN[12] (NHS GMS) pipelines. Variant interpretation was performed within an applied Genomics England PanelApp virtual gene panel (available at https://panelapp.genomicsengland.co.uk/) tailored to the individual's phenotype[13,14]. A.II-1 and A.II-3 were analyzed using the "posterior segment abnormalities" panel (v1.65; encompassing 174 genes), the "rare multisystem ciliopathy disorders" (v1.39; encompassing 77 genes) and the "significant early-onset obesity +/- other endocrine features and short stature" panel (v1.2; encompassing 21 genes). B.II-1 was analyzed using the "retinal disorders" panel (v2.195; encompassing 201 genes).

Individuals C.II-1 and C.II-2 and family members were sequenced using both Illumina short-read and Oxford Nanopore Technologies (ONT) GS with read coverage 30x and 10x respectively, alongside a commercial inherited retinal dystrophy (IRD) gene panel. Raw sequencing reads were aligned to the human reference genome using Burrows–Wheeler Aligner (BWA)[15] for short reads and Minimap2[16] for long reads. Variant calling was subsequently conducted using GATK4[17] for short-read data and Clair3[18] for long-read data to pinpoint SNVs and small INDELs. Structural variant (SV) and copy number variant (CNV) detection was carried out using DELLY[19], LUMPY[20], Manta[10], and CNVnator[21] for short-read data, and Sniffles2[22] for long-read data, with subsequent annotation using AnnotSV[23].

For Family D, DNA samples of the proband (Individual D.II-2) and family members were obtained from whole-blood or saliva samples. Exome sequencing was performed on the proband's DNA at CeGaT GmbH, (Tübingen, Germany). There, sequencing libraries were generated using the Twist Human Core Exome Plus kit (Twist Bioscience), following manufacturer's protocols. Libraries underwent paired-end sequencing on a Novaseq 6000 (Novogene, CeGaT) resulting in sequences of 100 bases. The average coverage was higher than 150X in targeted regions and resulting in ~90% of targeted regions with a coverage higher than 20X. Mapping, variant calling and variant annotation was performed using standard tools and in-house scripts as previously described[15]. This included the detection of SNVs, INDELs CNVs and mobile element insertions using HaplotypeCaller (GATK)[17], ExomeDepth[25], Scramble[26] and MELT[27]. The variants were confirmed by Sanger sequencing and co-segregation analysis was performed in all available family members.

To minimize the risk of inaccurate variant calls due to sequencing or alignment artefacts, all candidate variants were manually inspected using the Integrative Genomics Viewer (IGV)[28], comparing short- and long-read data where available.

Variant identification and prioritization were performed independently at the contributing institutions using comparable, clinically validated pipelines. In all cases, initial clinical-grade variant analysis in all affected individuals did not identify any pathogenic or potentially pathogenic genotypes in known IRD-associated genes listed in the Retinal Information Network (RetNet; https://retnet.org/)[29], nor in genes included in the Genomics England PanelApp virtual gene panel for IRD or other phenotype-driven panels relevant to each individual[9,10].

Research-based re-analysis: variant prioritization and interpretation

Subsequent research re-analysis focused on rare variants with a minor allele frequency of <0.001 in the gnomAD v4.1.0 population dataset (under the assumption that common variants are unlikely to be causative of rare disease), with additional filtering based on variant quality metrics and zygosity consistent with the inheritance pattern most compatible with the family structure. For Families A and C, in which two affected siblings were born to unaffected parents, analysis prioritized variants consistent with autosomal recessive inheritance (i.e. homozygous or compound heterozygous configurations). For singleton cases (Families B and D), a broader analysis was undertaken to include both monoallelic and biallelic rare variants, with inheritance models refined according to the segregation and phenotypic data available.

Predicted loss-of-function (nonsense, frameshift, canonical splice-site) and protein-altering missense variants were prioritized, as well as rare intronic variants predicted to affect splicing. Where relevant, in silico tools including REVEL, AlphaMissense, MutScore, SpliceAI, and Pangolin were used to support variant interpretation[30-34]; thresholds were not applied as hard filters but used in conjunction with gene-specific, segregation, and phenotypic evidence.

All novel variants were validated and annotated following HGVS nomenclature and interpreted using ACMG/AMP and ACGS guidelines[35,36].

Targeted Sanger sequencing

For Family D, Primer3Plus was used to design primers for polymerase chain reactions (PCR), performed using the GoTaq polymerase (Promega) and approximately 2ng of template DNA, according to the standard PCR cycling protocol. All PCR products were treated with ExoSAP-IT (Thermo Fisher) and Sanger sequencing was performed by Microsynth (Switzerland). Oligos used are CR-8251: atccccaaaaattcctcagc, CR-8252: cgcaatggcttctgcttatt, CR-8253: cagaggttccttccatgatttc, and CR-8254: aaagtccaccagtgtgattgtg.

Gene expression analysis

Violin plots were generated using the "sc.pl.violin" function from the SCANPY toolkit[18]. Gene expressions were derived from the published human retina cell atlas[19] and mouse retina cell atlas[20]. Expression distributions were visualized across annotated retinal major cell classes, as defined in the respective atlases.

RNA-seq data analysis

RNA-seq data was retrieved from Schumacker *et al*[6]. All samples for macula, peripheral retina and RPE/choroid were pooled and aligned to the reference genome using STAR with default parameters on hg19 reference genome. Reads spanning the analysed exons were quantified using IGV.

Animals

Wild-type C57BL/6 mice were purchased from the Jackson Labs (#000664). Animals were housed under a 12-h light/12-h dark cycle with access to water and food. All animal experimental procedures were approved by the Institutional Animal Care and Use Committee of the University of California, Irvine, and were performed in accordance with the NIH Guidelines for the Care and Use of Laboratory Animals.

Heterozygous Rd10 mice were purchased from the Jackson Labs (#004297). Mice were kept in individually ventilated cages (GM500, Tecniplast, Italy) with nesting material (3084001, Zoonlab, Germany) and bedding (Lignocel BK8-15, Rettenmaier & Söhne GmbH & Co KG, Germany). Mice were housed in groups (max. five animals per cage), maintained on a 12:12 light:dark cycle and water and food pellets (irradiated rodent diet 3302.PX.V20, Granovit AG, Switzerland) were provided ad libitum. Mice were deeply anesthetized with isoflurane (5% vol/vol, in 95% O2) and euthanized by decapitation. Eyes were enucleated with an angled forceps (F.S.T., #11051-10) and immediately transferred to ice-cold PBS solution (Thermo Fisher Scientific, #AM9625). After removing the cornea and the lens, vitrectomy was performed and the retina was dissected from the choroid/sclera using a Vannas-Tübingen spring scissors (F.S.T., #15003-08) and blunted-tip forceps (F.S.T., #11253-20).

Cell preparation and sorting

Mice retina were dissociated into single cells using the Neural Tissue Dissociation Kit P (Miltenyi Biotec), following the manufacturer's instructions. The resulting cell suspension was spun down for 5 min at 300×g in a pre-refrigerated centrifuge (Eppendorf). The supernatant was carefully removed without disturbing the pellet. Cells were strained through a 70- and then 40-μm filters (pluriSelect) and stained with propidium iodide (PI, 1 mg/ml, ThermoFisher Scientific) at room temperature to label dying cells.

FACSAria Fusion (100-μm nozzle, 20 psi) instrument equipped with FACSDiva software (v8.0.2, BD Biosciences) was used for excluding debris, doublets and PI+ cells. We employed a gating strategy that had been shown in-house to enrich for photoreceptor cells. LoBind twin.tec 384-well plates (Eppendorf) were used in all experiments.

Single cell RNA long read sequencing

Cells were sorted in individual wells containing 0.3 μl lysis buffer **(https://doi.org/10.1038/s41587-022-01312-3)** without oligodT was dispensed per well and the plate were stored at -80 °C until needed. Once ready for processing, 0.0055 μl of oligo-dT (variable sequence, 100 μM, IDT) were added per well with the I.DOT nanodispenser (Dispendix) directly after thawing. Samples were incubated for 3 min at 72 °C, placed on a metal block kept in an ice bucket for 5 minutes; 1.2 μl of FLASH-seq RT-PCR master mix was then added with the I-DOT and 21 PCR cycles were performed.

Following RT-PCR, the cDNA was diluted 1:10 with RNAse-free water using the I.DOT. The Fluent 780 workstation was used to transfer 1.5 µl diluted cDNA into a new 384-well plate.

Cell barcoding primers were obtained from IDT and diluted to 0.4 µM or 2 µM in a 384-well plate. Using the Fluent 780 workstation, 1.25 µl of barcoding primer was added to each cell at a 0.4 µM dilution. The PCR master mix was assembled using the KAPA HiFi HotStart PCR Kit (Roche) and dispensed with the I.DOT (0.7 µl 5x KAPA HiFi Buffer, 0.15 µl dNTP [10 mM], 0.1 µl KAPA HiFi HotStart DNA Polymerase [1 U/µl] and 0.8 µl RNAse-free water). The following PCR program was used: 98 °C for 3 min, then 8 cycles of (98 °C for 20 s, 63 °C for 15 s, 72 °C for 5 min). After PCR amplification, the cDNAs were collected and pooled. Magnetic bead purification was performed with 0.8:1 ratio of SeraMag Beads (GE Healthcare) containing 18% w/v polyethylene glycol (molecular weight = 8,000) (Sigma-Aldrich). Of note, the first incubation of beads with cDNA was done for 10 minutes at RT. Two washes with 80% ethanol were performed. The cDNA was eluted in 40 µl of nuclease-free water. The concentration and library sizes were measured on a Qubit Fluorometer (HS dsDNA assay) and 2100 Bioanalyzer System (HS DNA chip, software vB.02.10.51764, Agilent), respectively.

The Ligation Sequencing Kit V14 (SQK-LSK114, ONT) was used to prepare the final library. When multiplexing samples, equivalent concentrations were used. We started the library preparation with >150 fmol. The 0.4:1 magnetic beads:DNA ratio recommended by ONT was replaced with 0.8:1. The Short Fragment Buffer was used. We loaded ~12-15 fmol of the library on the chip.

Cell demultiplexing

Cell barcoding detection and demultiplexing was performed using the FSNanoporeR pipeline (https://github.com/vincenthahaut/IOB/tree/master/6_Nanopore-FS/FSnanoporeR). FSNanoporeR pipeline uses BLAST-short (v2.14.0) to detect PCR adapter sequences ("ISPCR", AAGCAGTGGTATCAACGCAGAGT) (-strand plus -word_size 11 -gapopen 1 -gapextend 1 -window_size 0 -perc_identity 75). Reads displaying >2 ISPCR sequences or any ISPCR at >200 bp of the read edges were marked as chimeric reads. The positions of the TSO and oligo-dT were also identified in each read. The TSO, oligo-dT, and the UMI were subsequently trimmed from the reads, and the UMI sequence was extracted for downstream quantification.

Then, barcode sequences were extracted using vsearch (v2.22.1), searching for the PCR-anchor ISPCR (CAGCACCTCGACGCTCTTCCGATCT NNNNNNNNNNNNN AAGCAGTGGTATCAACGCAGAGT) sequence (--maxaccepts 5 --strand plus --wordlength 3 --minwordmatches 12 --mincols 40 -id 0.7) in either the first or last 200 bp of the reads (seqkit subseq, v2.4.0). To assign each read to a cell, barcodes at the start and end of the read were compared against a whitelist of known barcodes. Barcodes with sequencing errors were corrected by finding the closest unique match in the whitelist based on Levenshtein distance. A read was assigned to a specific cell barcode only if a unique match could be determined. Reads that could not be unambiguously assigned to a single barcode were marked as 'Undetermined'.

Reference Genomes

Mouse data were mapped onto GRCm38 (Gencode v23, primary assembly). Corresponding gencode annotations were used.

Mapping and read assignment

Read mapping and assignment was also performed with the FSNanoporeR pipeline. Here, demultiplexed reads were mapped onto the reference genome with minimap2 (v2.26-r1175) in splice-aware mode (*-ax-splice*), providing the annotation (*--junc-bed*). Samtools *view* (v1.15.1) was used to select primary mappings (*-F 2308*). Isoquant (v3.2.0) was used to assign reads to features (*--count_exons --data_type nanopore --stranded none*). Reads associated with multiple transcripts were systematically discarded. The generated BAM files were then parsed with Umi-tools (v1.1.4) to deduplicate reads per gene and cell barcode and the final results were collapsed into count matrices.

Cell type identification

Gene count files were consolidated into a single gene-by-cell count matrix for each sample. The resulting count matrices were then merged under one Seurat object and cells with fewer than 200 detected genes expressed or with mitochondrial gene content exceeding 15% were filtered out.

The filtered data was log-normalized and the top 2,000 most highly variable genes (HVGs) were identified using the variance-stabilizing transformation. The expression levels of these HVGs were scaled across all cells. Principal Component Analysis (PCA) was performed on the scaled HVGs and a shared nearest neighbour (SNN) graph was constructed using the first 10 PCs. Clusters were identified by applying the Louvain algorithm with a resolution of 0.1. Clusters were manually annotated based on the expression of known canonical marker genes for major retinal cell types. These included Rho and Gngt1 (Rods), Arr3 (Cones), Apoe (Müller Glia), and Prkca, Trpm1, Grik1, and Prdm8 (Bipolar Cells). Following annotation, the cell IDs for each identified cell type were exported and samtools was used to generate a cell-type specific BAM file for downstream analysis.

Transcript isoform discovery and quantification using Oxford Nanopore Technologies long-read single-cell RNA-seq data in adult human retina (macula)

Following a similar approach as described in Wang *et al*[21], we extracted total cDNA from macular tissues collected from 2 donors and prepared libraries following the Nanopore single-cell transcriptomics protocol (10x Genomics, version Jan 2022) with the SQK-PCS111 Ligation Sequencing Kit. Sequencing was performed on PromethION FLO-PRO002 R9.4.1 flow cells. Base calling of raw ONT data was performed using Guppy (v6.1.5). Long-read data preprocessing - including quality control, alignment, cell barcode and unique molecular identifier (UMI) correction, and demultiplexing - was conducted using the single-cell workflow from EPI2ME Labs (https://github.com/epi2me-labs/wf-single-cell). Cell-class annotation was derived from the short-read data using shared barcodes with the long-read dataset. Transcript isoform identification, classification, and quantification were carried out using FLAIR[22] and SQANTI3[23], focusing on reads aligned to the *FSD1L* locus in cone and rod photoreceptor cell classes. Based on the quantification of transcript isoform, we calculated the usage of each exon.

Minigene assay

To evaluate the effect of the intronic variant, we constructed variant specific minigenes, according to the procedure described previously[24]. Briefly, we designed primers with attB sites that were binding 829bp downstream and 343bp upstream of the 26bp intronic deletion (CR-9251: 5'-ggggacaagtttgtacaaaaaagcaggcttgtggtgatagctgtgtttca-3' and CR-9252: 5'-ggggaccactttgtacaagaaagctgggtgccaggtatgagtaaaagcagaa-3'; the underlined sequences designating attB1 and attB2 tails). The region with the variant and the wild-type one were amplified from the compound heterozygous proband (Individual D:II-2, CHlaus0427) DNA and anonymous control using GoTaq G2 DNA polymerase. The gel-purified PCR products were first ligated into the pGEM-T Easy vector (Promega, Madison, Wisconsin, USA) using T4 DNA ligase. The ligated plasmids were transformed into in-house prepared Stbl3 competent cells via electroporation. The recovered bacterial culture was plated on ampicillin (100 µg/ml) agar plates with blue-white selection. White-colonies (positive for the insert) were further verified by Sanger sequencing using Microsynth NightSeq service. The correctly cloned plasmids were purified and were used to shuttle the inserts into pDONR201 (Thermo Fisher Scientific Inc., Carlsbad, California, USA) using BP clonase and finally into pCI-NEO-RHO exon3,5/DEST using LR Clonase[25]. The final plasmids before transfection were validated with Sanger sequencing.

ARPE-19 cells were cultured in DMEM/F-12 (Dulbecco's Modified Eagle Medium/Nutrient Mixture F-12, Gibco, 31331-028) and 10% fetal bovine serum (Sigma, F7524). All cells were grown at 37 °C in a humidified incubator maintained at 5% CO2.

ARPE-19 cells were transfected with wt and mutant *FSD1L* minigenes (c.1025+624_1025+649del), untransfected cells were used as control. Per condition, 3×10^5 cells were seeded in 6-well plates. The cells were transfected after 24 h using the FuGENE HD transfection reagent (Promega) according to manufacturer's instructions and using a µl FuGene reagent:µg DNA ratio of 3:1. Total RNA was extracted 24 h after transfection with RNAspin Mini Kit (Cytiva) and reverse transcribed using High-Capacity cDNA Reverse Transcription Kit into cDNA in 20 µl reactions containing 1ug of RNA. PCR was performed on 1 µl cDNA using pCI-neoRHO specific primers CR-8303 and CR-8304 and KAPA HiFi DNA Polymerase. The PCR products were separated on 2% agarose gels containing GelRed Nucleic Acid Stain and visualized under UV-light.

PBMCs isolation and cultivation

Peripheral blood mononuclear cells were isolated after peripheral blood drawing in BD vacutainer containing acid citric dextrose (ACD) solution A from Becton Dickinson. Blood was diluted in phosphate buffered saline (PBS, 1:1) and layered into lymphocyte separation medium 1077 from Merck (1:1). Following a centrifugation for 30 minutes at 400g, plasma was removed and interface (2ml) was collected and diluted in 20ml serum-free RPMI1640 medium (Thermo Fisher). Following a centrifugation for 10 minutes at 200g and removal of the supernatant, the pellet was resuspended in 1ml preservation medium (RPMI, 10% DMSO and 10% fetal bovine serum from Sigma, F7524). Cells were counted and frozen at -80C first, and -140C later.

RNA extraction from PBMCs and cDNA synthesis

Frozen PBMCs were thawed and cultivated for at least 24h in RPMI1640 medium supplemented with 10-15% fetal bovine serum (Sigma, F7524) at 37 °C in a humidified incubator maintained at 5% CO2.

RNA was extracted from PBMCs using the RNAspin Mini RNA Isolation Kit from Cytiva and 200ng were converted into cDNA with the High capacity cDNA Reverse Transcription kit (Thermo Fisher). PCR on the cDNA was performed using standard cycling conditions and the following primers CR-8265: GGATAACTCCTCATCCCATTTG and CR-9231: AAGGCCACCTAATCAAGACG, leading to a 728bp expected product.

Ultrastructure expansion microscopy (U-ExM)
Human retina was isolated following autopsy with fixation, gelation, staining, and expansion performed exactly as laid out[8], the only changes pertained to antibodies/dilutions (Primary antibodies: Anti-FSD1L: ProteinTech Group Cat# 21032-1-AP, 5 µg; anti-FSD1L: ThermoFisher, Cat# BS-13221R, 5 µg; anti-a-tubulin: ABCD Antibodies, Cat# AA345, 1:100; anti-b-tubulin: ABCD Antibodies, Cat# AA344, 1:100; anti-rhodopsin: Merck Cat# MAB5356, 1:1000; Secondary antibodies, 1:500 used for all: Alexa Fluor 488 F(ab')2 fragment of goat anti-rabbit IgG, ThermoFisher A-11070; F(ab')2-Goat anti-Mouse IgG (H+L) Cross-Adsorbed Secondary Antibody, Alexa Fluor™ 555, ThermoFisher A-21425; Anti-Guinea Pig IgG (H+L), CF™ 647 antibody produced in goat, Sigma SAB4600180). An expansion factor of ~4x was obtained (gelled controls displayed in Supplemental Figure S4). Imaging was performed using 35mm glass bottom dishes with a 10mm microwell (MatTek Life Sciences, Cat# P35G-1.5-14-C, Ashland, Massachusetts, USA) that had been coated in Poly-L-lysine. A gel slice was placed on the dish, a drop of water added, and coverslip added on top. The imaging was performed on a Leica Stellaris 8 Falcon using HyD lasers and a 63x HC PL APO oil immersion objective, NA 1.40, with an optical zoom between 3-7.

Supplemental acknowledgements

This research was made possible through access to data in the National Genomic Research Library, which is managed by Genomics England Limited (a wholly owned company of the Department of Health and Social Care). The National Genomic Research Library holds data provided by individuals and collected by the NHS as part of their care and data collected as part of their participation in research. The National Genomic Research Library is funded by the National Institute for Health Research and NHS England. The Wellcome Trust, Cancer Research UK and the Medical Research Council have also funded research infrastructure.

Supplemental references

1. Lindeboom, R.G., Supek, F., and Lehner, B. (2016). The rules and impact of nonsense-mediated mRNA decay in human cancers. Nat Genet *48*, 1112-1118. 10.1038/ng.3664.
2. Nagy, E., and Maquat, L.E. (1998). A rule for termination-codon position within intron-containing genes: when nonsense affects RNA abundance. Trends Biochem Sci *23*, 198-199. 10.1016/s0968-0004(98)01208-0.
3. Stein, P.A., Toret, C.P., Salic, A.N., Rolls, M.M., and Rapoport, T.A. (2002). A novel centrosome-associated protein with affinity for microtubules. J Cell Sci *115*, 3389-3402. 10.1242/jcs.115.17.3389.
4. Mirdita, M., Schutze, K., Moriwaki, Y., Heo, L., Ovchinnikov, S., and Steinegger, M. (2022). ColabFold: making protein folding accessible to all. Nat Methods *19*, 679-682. 10.1038/s41592-022-01488-1.
5. Jumper, J., Evans, R., Pritzel, A., Green, T., Figurnov, M., Ronneberger, O., Tunyasuvunakool, K., Bates, R., Zidek, A., Potapenko, A., et al. (2021). Highly accurate protein structure prediction with AlphaFold. Nature *596*, 583-589. 10.1038/s41586-021-03819-2.
6. Schumacker, S.T., Coppage, K.R., and Enke, R.A. (2020). RNA sequencing analysis of the human retina and associated ocular tissues. Sci Data *7*, 199. 10.1038/s41597-020-0541-4.
7. Garcia-Perez, R., Ramirez, J.M., Ripoll-Cladellas, A., Chazarra-Gil, R., Oliveros, W., Soldatkina, O., Bosio, M., Rognon, P.J., Capella-Gutierrez, S., Calvo, M., et al. (2023). The landscape of expression and alternative splicing variation across human traits. Cell Genom *3*, 100244. 10.1016/j.xgen.2022.100244.
8. Mercey, O., Kostic, C., Bertiaux, E., Giroud, A., Sadian, Y., Gaboriau, D.C.A., Morrison, C.G., Chang, N., Arsenijevic, Y., Guichard, P., and Hamel, V. (2022). The connecting cilium inner scaffold provides a structural foundation that protects against retinal degeneration. PLoS Biol *20*, e3001649. 10.1371/journal.pbio.3001649.
9. Raczy, C., Petrovski, R., Saunders, C.T., Chorny, I., Kruglyak, S., Margulies, E.H., Chuang, H.Y., Kallberg, M., Kumar, S.A., Liao, A., et al. (2013). Isaac: ultra-fast whole-genome secondary analysis on Illumina sequencing platforms. Bioinformatics *29*, 2041-2043. 10.1093/bioinformatics/btt314.
10. Chen, X., Schulz-Trieglaff, O., Shaw, R., Barnes, B., Schlesinger, F., Kallberg, M., Cox, A.J., Kruglyak, S., and Saunders, C.T. (2016). Manta: rapid detection of structural variants and indels for germline and cancer sequencing applications. Bioinformatics *32*, 1220-1222. 10.1093/bioinformatics/btv710.
11. Roller, E., Ivakhno, S., Lee, S., Royce, T., and Tanner, S. (2016). Canvas: versatile and scalable detection of copy number variants. Bioinformatics *32*, 2375-2377. 10.1093/bioinformatics/btw163.
12. Behera, S., Catreux, S., Rossi, M., Truong, S., Huang, Z., Ruehle, M., Visvanath, A., Parnaby, G., Roddey, C., Onuchic, V., et al. (2025). Comprehensive genome analysis and variant detection at scale using DRAGEN. Nat Biotechnol *43*, 1177-1191. 10.1038/s41587-024-02382-1.
13. The National Genomic Research Library v5.1. (2020). https://doi.org/10.6084/m9.figshare.4530893.v7.

14. Martin, A.R., Williams, E., Foulger, R.E., Leigh, S., Daugherty, L.C., Niblock, O., Leong, I.U.S., Smith, K.R., Gerasimenko, O., Haraldsdottir, E., et al. (2019). PanelApp crowdsources expert knowledge to establish consensus diagnostic gene panels. Nat Genet *51*, 1560-1565. 10.1038/s41588-019-0528-2.
15. Li, H., and Durbin, R. (2009). Fast and accurate short read alignment with Burrows-Wheeler transform. Bioinformatics *25*, 1754-1760. 10.1093/bioinformatics/btp324.
16. Li, H. (2018). Minimap2: pairwise alignment for nucleotide sequences. Bioinformatics *34*, 3094-3100. 10.1093/bioinformatics/bty191.
17. McKenna, A., Hanna, M., Banks, E., Sivachenko, A., Cibulskis, K., Kernytsky, A., Garimella, K., Altshuler, D., Gabriel, S., Daly, M., and DePristo, M.A. (2010). The Genome Analysis Toolkit: a MapReduce framework for analyzing next-generation DNA sequencing data. Genome Res *20*, 1297-1303. 10.1101/gr.107524.110.
18. Zheng, Z., Li, S., Su, J., Leung, A.W., Lam, T.W., and Luo, R. (2022). Symphonizing pileup and full-alignment for deep learning-based long-read variant calling. Nat Comput Sci *2*, 797-803. 10.1038/s43588-022-00387-x.
19. Rausch, T., Zichner, T., Schlattl, A., Stutz, A.M., Benes, V., and Korbel, J.O. (2012). DELLY: structural variant discovery by integrated paired-end and split-read analysis. Bioinformatics *28*, i333-i339. 10.1093/bioinformatics/bts378.
20. Layer, R.M., Chiang, C., Quinlan, A.R., and Hall, I.M. (2014). LUMPY: a probabilistic framework for structural variant discovery. Genome Biol *15*, R84. 10.1186/gb-2014-15-6-r84.
21. Abyzov, A., Urban, A.E., Snyder, M., and Gerstein, M. (2011). CNVnator: an approach to discover, genotype, and characterize typical and atypical CNVs from family and population genome sequencing. Genome Res *21*, 974-984. 10.1101/gr.114876.110.
22. Smolka, M., Paulin, L.F., Grochowski, C.M., Horner, D.W., Mahmoud, M., Behera, S., Kalef-Ezra, E., Gandhi, M., Hong, K., Pehlivan, D., et al. (2024). Detection of mosaic and population-level structural variants with Sniffles2. Nat Biotechnol *42*, 1571-1580. 10.1038/s41587-023-02024-y.
23. Geoffroy, V., Herenger, Y., Kress, A., Stoetzel, C., Piton, A., Dollfus, H., and Muller, J. (2018). AnnotSV: an integrated tool for structural variations annotation. Bioinformatics *34*, 3572-3574. 10.1093/bioinformatics/bty304.
24. Peter, V.G., Kaminska, K., Santos, C., Quinodoz, M., Cancellieri, F., Cisarova, K., Pescini Gobert, R., Rodrigues, R., Custodio, S., Paris, L.P., et al. (2023). The first genetic landscape of inherited retinal dystrophies in Portuguese patients identifies recurrent homozygous mutations as a frequent cause of pathogenesis. PNAS Nexus *2*, pgad043. 10.1093/pnasnexus/pgad043.
25. Plagnol, V., Curtis, J., Epstein, M., Mok, K.Y., Stebbings, E., Grigoriadou, S., Wood, N.W., Hambleton, S., Burns, S.O., Thrasher, A.J., et al. (2012). A robust model for read count data in exome sequencing experiments and implications for copy number variant calling. Bioinformatics *28*, 2747-2754. 10.1093/bioinformatics/bts526.
26. Torene, R.I., Galens, K., Liu, S., Arvai, K., Borroto, C., Scuffins, J., Zhang, Z., Friedman, B., Sroka, H., Heeley, J., et al. (2020). Mobile element insertion detection in 89,874 clinical exomes. Genet Med *22*, 974-978. 10.1038/s41436-020-0749-x.

27. Gardner, E.J., Lam, V.K., Harris, D.N., Chuang, N.T., Scott, E.C., Pittard, W.S., Mills, R.E., Genomes Project, C., and Devine, S.E. (2017). The Mobile Element Locator Tool (MELT): population-scale mobile element discovery and biology. Genome Res *27*, 1916-1929. 10.1101/gr.218032.116.
28. Robinson, J.T., Thorvaldsdottir, H., Winckler, W., Guttman, M., Lander, E.S., Getz, G., and Mesirov, J.P. (2011). Integrative genomics viewer. Nat Biotechnol *29*, 24-26. 10.1038/nbt.1754.
29. Daiger, S., Rossiter, B., Greenberg, J., Christoffels, A., and Hide, W. (1998). Data services and software for identifying genes and mutations causing retinal degeneration. Invest Ophthalmol Vis Sci *39*.
30. Cheng, J., Novati, G., Pan, J., Bycroft, C., Zemgulyte, A., Applebaum, T., Pritzel, A., Wong, L.H., Zielinski, M., Sargeant, T., et al. (2023). Accurate proteome-wide missense variant effect prediction with AlphaMissense. Science *381*, eadg7492. 10.1126/science.adg7492.
31. Ioannidis, N.M., Rothstein, J.H., Pejaver, V., Middha, S., McDonnell, S.K., Baheti, S., Musolf, A., Li, Q., Holzinger, E., Karyadi, D., et al. (2016). REVEL: An Ensemble Method for Predicting the Pathogenicity of Rare Missense Variants. Am J Hum Genet *99*, 877-885. 10.1016/j.ajhg.2016.08.016.
32. Jaganathan, K., Kyriazopoulou Panagiotopoulou, S., McRae, J.F., Darbandi, S.F., Knowles, D., Li, Y.I., Kosmicki, J.A., Arbelaez, J., Cui, W., Schwartz, G.B., et al. (2019). Predicting Splicing from Primary Sequence with Deep Learning. Cell *176*, 535-548 e524. 10.1016/j.cell.2018.12.015.
33. Quinodoz, M., Peter, V.G., Cisarova, K., Royer-Bertrand, B., Stenson, P.D., Cooper, D.N., Unger, S., Superti-Furga, A., and Rivolta, C. (2022). Analysis of missense variants in the human genome reveals widespread gene-specific clustering and improves prediction of pathogenicity. Am J Hum Genet *109*, 457-470. 10.1016/j.ajhg.2022.01.006.
34. Zeng, T., and Li, Y.I. (2022). Predicting RNA splicing from DNA sequence using Pangolin. Genome Biol *23*, 103. 10.1186/s13059-022-02664-4.
35. Durkie, M., Cassidy, E.-J., Berry, I., Owens, M., Turnbull, C., Scott, R.H., Taylor, R.W., Deans11, Z.C., Ellard, S., and Baple, E.L. (2023). ACGS Best Practice Guidelines for Variant Classification in Rare.
36. Richards, S., Aziz, N., Bale, S., Bick, D., Das, S., Gastier-Foster, J., Grody, W.W., Hegde, M., Lyon, E., Spector, E., et al. (2015). Standards and guidelines for the interpretation of sequence variants: a joint consensus recommendation of the American College of Medical Genetics and Genomics and the Association for Molecular Pathology. Genet Med *17*, 405-424. 10.1038/gim.2015.30.
37. Wolf, F.A., Angerer, P., and Theis, F.J. (2018). SCANPY: large-scale single-cell gene expression data analysis. Genome Biol *19*, 15. 10.1186/s13059-017-1382-0.
38. Li, J., Wang, J., Ibarra, I.L., Cheng, X., Luecken, M.D., Lu, J., Monavarfeshani, A., Yan, W., Zheng, Y., Zuo, Z., et al. (2023). Integrated multi-omics single cell atlas of the human retina. Res Sq. 10.21203/rs.3.rs-3471275/v1.
39. Li, J., Choi, J., Cheng, X., Ma, J., Pema, S., Sanes, J.R., Mardon, G., Frankfort, B.J., Tran, N.M., Li, Y., and Chen, R. (2024). Comprehensive single-cell atlas of the mouse retina. iScience *27*, 109916. 10.1016/j.isci.2024.109916.
40. Wang, M., Li, Y., Wang, J., Oh, S.H., Cao, Y., and Chen, R. (2025). Integrating short-read and long-read single-cell RNA sequencing for comprehensive

transcriptome profiling in mouse retina. Genome Res *35*, 740-754. 10.1101/gr.279167.124.

41. Tang, A.D., Soulette, C.M., van Baren, M.J., Hart, K., Hrabeta-Robinson, E., Wu, C.J., and Brooks, A.N. (2020). Full-length transcript characterization of SF3B1 mutation in chronic lymphocytic leukemia reveals downregulation of retained introns. Nat Commun *11*, 1438. 10.1038/s41467-020-15171-6.
42. Tardaguila, M., de la Fuente, L., Marti, C., Pereira, C., Pardo-Palacios, F.J., Del Risco, H., Ferrell, M., Mellado, M., Macchietto, M., Verheggen, K., et al. (2018). SQANTI: extensive characterization of long-read transcript sequences for quality control in full-length transcriptome identification and quantification. Genome Res *28*, 396-411. 10.1101/gr.222976.117.
43. Ullah, M., Rehman, A.U., Quinodoz, M., Rashid, A., Cancellieri, F., Munir, A., Kaminska, K., Iqbal, A., Javed, S., Dawood, M., et al. (2025). A comprehensive genetic landscape of inherited retinal diseases in a large Pakistani cohort. NPJ Genom Med *10*, 31. 10.1038/s41525-025-00488-2.
44. Sangermano, R., Khan, M., Cornelis, S.S., Richelle, V., Albert, S., Garanto, A., Elmelik, D., Qamar, R., Lugtenberg, D., van den Born, L.I., et al. (2018). ABCA4 midigenes reveal the full splice spectrum of all reported noncanonical splice site variants in Stargardt disease. Genome Res *28*, 100-110. 10.1101/gr.226621.117.
